# The physicochemical basis of protein evolution: property-informed evolutionary models (PRIME)

**DOI:** 10.1093/molbev/msag200

**Published:** 2026-08-13

**Authors:** Hannah Kim, Konrad Scheffler, Anton Nekrutenko, Darren P Martin, Steven Weaver, Ben Murrell, Sergei L Kosakovsky Pond

**Affiliations:** Institute for Genomics and Evolutionary Medicine, Temple University, Philadelphia, PA, USA; Department of Biology, Temple University, Philadelphia, PA, USA; Department of Biochemistry and Molecular Biology, The Pennsylvania State University, University Park, PA, USA; Institute of Infectious Disease and Molecular Medicine, University of Cape Town, Cape Town, South Africa; Institute for Genomics and Evolutionary Medicine, Temple University, Philadelphia, PA, USA; Department of Microbiology, Tumor and Cell Biology, Karolinska Institutet, Stockholm, Sweden; Institute for Genomics and Evolutionary Medicine, Temple University, Philadelphia, PA, USA; Department of Biology, Temple University, Philadelphia, PA, USA

**Keywords:** codon substitution models, physicochemical properties, protein evolution, natural selection, maximum likelihood phylogenetics

## Abstract

Standard probabilistic models of coding sequence evolution effectively identify where and when selection acts but remain agnostic to the mechanistic realization of these forces. We introduce PRIME (PRoperty Informed Models of Evolution), a framework of codon-level maximum likelihood methods—including global (G-PRIME), episodic (E-PRIME), and site-specific (S-PRIME) implementations—that explicitly model amino acid exchangeability as a function of physicochemical properties. By parameterizing attributes such as molecular volume, hydropathy, and secondary structure propensities, PRIME aims to resolve the biophysical basis of selective constraint across both the sequence and the phylogeny. At the site level, S-PRIME leverages an explicit biophysical taxonomy to categorize residues as conserved, neutral, or changing for specific properties, resolving selective signals that are missed by traditional rate-based metrics. Our analysis of a benchmark of 24 diverse datasets and a genome-wide screen of 18,944 mammalian genes demonstrates that consideration of biophysical realism can yield substantial improvements in model fit, acting synergistically with rate variation to explain complex evolutionary patterns. We find that physicochemical constraints at individual sites can be reliably detected in datasets with sufficient information redundancy (substitutions per unique amino acid; AUC=0.91), with sensitivity exceeding 90% in data-rich alignments. E-PRIME reveals a distinct hierarchy in biophysical constraints: while core packing and beta-sheet scaffolds are rigidly conserved, alpha-helix propensity and surface electrostatics serve as the primary substrates for adaptive tuning. Furthermore, PRIME importance weights align with aspects of the primary semantic axes of deep learning representations (ESM-2) and capture key features of experimental fitness landscapes. By transforming abstract evolutionary rates into interpretable biophysical rules, PRIME provides a useful framework for characterizing the mechanistic drivers of protein diversity.

## Introduction

Deciphering the selective pressures acting on viral genomes and other organisms requires robust probabilistic models of protein evolution (Durbin et al. 1998; Felsenstein 2004; Yang 2006). These statistical tools are indispensable for investigating phenomena such as host switching (Ngandu et al. 2009), immune response (Delport et al. 2008; Lacerda et al. 2010), and the emergence of drug resistance (Seoighe et al. 2007; Kosakovsky Pond et al. 2008; Murrell, de Oliveira et al. 2012). Yet, while current methodologies have achieved high sensitivity in detecting *where* in the sequence (Murrell, Wertheim, Moola et al. 2012) and *when* in evolutionary history (Murrell et al. 2015) selection acts, they often fail to explain *how* this action is realized biochemically. A major gap remains in moving from the phenomenological detection of selection to the mechanistic characterization of the physicochemical rules that govern it.

The integration of physicochemical realism into codon substitution models traces its lineage to the seminal work of Grantham (1974), who introduced a composite metric of amino acid differences based on composition, polarity, and volume. This concept was subsequently adapted by Miyata et al. (1979) and formalized by Goldman and Yang (1994), who scaled substitution rates using these fixed distances. Xia and Li (1998) further demonstrated that the genetic code itself minimizes polarity and hydropathy changes. Earlier attempts to integrate physicochemical properties into likelihood frameworks include the site-heterogeneous amino acid models of Koshi and Goldstein (1998) and the distance-dependent codon models of Yang (2000). Subsequent approaches attempted to categorize substitutions as property-altering or conserving (Sainudiin et al. 2005; Wong et al. 2006), a concept recently refined by the CoRa model’s distinction between conservative and radical changes (Weber and Whelan 2019). Jones et al. (2020) introduced the phenotype-genotype branch-site model (PG-BSM), which explicitly links adaptive codon evolution to discrete phenotypic changes by detecting transient elevations in nonsynonymous rates, notably without requiring the nonsynonymous/synonymous rate ratio ω>1. Others, such as the LCAP model (Conant et al. 2007), sought to estimate property weights directly from data, though often at the cost of computational tractability or site-specific resolution. Despite these methodological advances, none have achieved broad adoption; the vast majority of evolutionary analyses continue to rely on standard *ω* models agnostic of the amino-acids being exchanged, possibly because these alternatives lack the intuitive appeal of a simple rate ratio, have not been supported by robust, scalable software implementations, and have not been shown to significantly impact biologically meaningful inference.

Simultaneously, biophysical studies have underscored that evolutionary trajectories are shaped by fundamental physical constraints. Seminal work by Bloom, Wilke, and colleagues demonstrated that protein evolution is often governed by a global requirement to maintain thermodynamic stability, leading to a marginal stability regime where most mutations are destabilizing but neutral as long as the protein remains folded (Bloom et al. 2006, 2007). Mirny and Shakhnovich (1999) similarly showed that conserved positions often serve as nuclei for folding. These biophysical constraints—including folding kinetics and solubility—act as a universal filter on sequence space (Liberles et al. 2012; Serohijos and Shakhnovich 2014). Furthermore, stability can promote evolvability by buffering the destabilizing effects of functionally innovative mutations (Tokuriki and Tawfik 2009). However, the implicit assumption that protein evolution is primarily constrained by a rigid 3D fold constitutes a significant limitation for many existing approaches. A substantial fraction of the proteome consists of Intrinsically Disordered Regions (IDRs) and flexible linkers which lack stable tertiary structure yet play vital roles in signaling and regulation (Dunker et al. 2001). Evolution in these regions is distinct, often driven by the conservation of distributed features—such as net charge, hydropathy, or flexibility—rather than strict site-specific identities (Zarin et al. 2019). Standard substitution models, and even those grounded in structural thermodynamics, may fail to capture the relaxed or orthogonal constraints acting on these flexible segments.

In the past several years, the field has been transformed by deep learning approaches, including AlphaFold (Jumper et al. 2021) and protein language models (PLMs) like ESM (Rives et al. 2021; Lin et al. 2023). These models have been highly effective in learning the implicit grammar of protein sequences, effectively capturing high-order dependencies and structural constraints. However, they fundamentally trade mechanistic interpretability for predictive power. While a PLM might accurately predict *that* a specific substitution is unlikely, it operates as a high-dimensional black box, obscuring *why* that constraint exists. Whether a site is conserved due to electrostatic requirements, packing density, or an unknown functional dependency remains opaque. Consequently, the lack of an explicit biophysical grammar limits our ability to predict the impact of novel mutations, particularly in contexts like immune escape where viruses explore regions of sequence space not well-represented in training data.

To bridge this divide, we introduce PRIME (PRoperty Informed Models of Evolution), a comprehensive family of probabilistic models that filters evolutionary action through the prism of physicochemical properties. We present three complementary implementations: a G-PRIME (Global PRIME) model for characterizing gene-wide constraints, an E-PRIME (Episodic PRIME) framework for detecting episodic property selection, and an S-PRIME (Site-level PRIME) inference method for resolving residue-specific biophysics. By encoding the intuition that properties like polarity and volume are conserved or driven to change, we estimate exchangeabilities within a maximum likelihood framework. This approach yields a marked improvement in model fit and provides a formal, standalone platform for testing biophysical hypotheses. While PRIME can assist in decoding the latent constraints of deep learning models, its primary power lies in its ability to help interpret the biochemical mechanisms of evolution—transforming abstract substitution rates into concrete physical rules.

## Methods

### Model description

We use the standard continuous-time Markov description of coding-sequence evolution (Goldman and Yang 1994; Muse and Gaut 1994). Key parameters and symbols used throughout the PRIME models are summarized in Table 1. The transition matrix for finite time t≥0 is given by $e^{Qt}$; the rate matrix $Q=\{q_{ij}\}$ describes the instantaneous rate of substitution of codon *i* with codon *j*:


(1) $$q_{ij}=\left\{ \begin{array}{cc} \alpha \theta_{ij}\pi_{\;j} & \;\text{for }\delta (i,j)=1,AA(i)=AA(j),\\ \beta_{xy}\theta_{ij}\pi_{\;j} & \;\text{for }\delta (i,j)=1,AA(i)=x\neq AA(j)=y,\\ 0 & \;\text{for }\delta (i,j)>1,\\ -\sum_{k\neq i}q_{ik} & \;\text{for }\delta (i,j)=0, \end{array}\right.$$

where δ(i,j) counts the number of nucleotide differences between codons *i* and *j*, and AA(i) stands for the amino acid corresponding to codon *i*. $\theta_{ij}$ are the nucleotide-level mutation parameters implementing the general time reversible (GTR) model for nucleotide substitution biases, with the rate of transitions between Adenine and Guanine set to 1 to ensure model identifiability. $\pi_{\;j}$ are equilibrium codon frequencies. *α* is a multiplier for synonymous substitutions. $\beta_{xy}$ is a multiplier for nonsynonymous substitutions representing the exchangeability of amino acids *x* and *y*.

**Table 1:** Key parameters and symbols used in PRIME models.

Symbol	Description
*α*	Synonymous substitution rate multiplier
$\beta_{xy}$	Nonsynonymous substitution rate multiplier between amino acids *x* and *y*
*ψ*	Baseline log nonsynonymous/synonymous rate ratio
*D*	Number of modeled physicochemical properties (e.g. D=5 for 5-property models)
$\lambda_{i}$	Importance coefficient for the *i*th physicochemical property
$x_{i}$	Value of the *i*th property for amino acid *x* (e.g. $x_{2}=\text{value}$ of property 2, volume, for amino acid *x*)

Parameters such as *α*, *ψ*, and *λ* may vary across sites and/or branches depending on the specific model configuration (global vs episodic vs site).

We depart from the standard model in that the nonsynonymous substitution rate depends on the amino acids involved in the substitution. Given a set of *D* properties such that the value of property *i* for amino acid *x* is $x_{i}$, we model $\beta_{xy}$ as:


(2) $$\beta_{xy,x\neq y}=\alpha^{\left(s\right)}\exp\;\left[\psi^{\left(s\right)}-\sum_{i=1}^{D}\lambda_{i}^{\left(s\right)}|x_{i}-y_{i}|\right]$$

Here, $\psi^{\left(s\right)}$ represents the baseline log nonsynonymous/synonymous rate ratio at site *s*. This formulation ensures that if all property weights $\lambda_{i}^{\left(s\right)}=0$, the model reduces to the standard MG94 model with $\omega_{s}=\exp\;(\psi^{\left(s\right)})$. The parameters $\lambda_{i}^{\left(s\right)}$ represent the importance of property *i* at site *s*. Positive values indicate conservation (purifying selection), where exchangeability declines as property difference increases. Negative values ($\lambda_{i}^{\left(s\right)}<0$) imply positive selection, where it is important for a property to change.

This general framework is deployed across three distinct implementations to capture selective constraints at different scales:

1. G-PRIME: Assumes constant *λ* values across the entire alignment, estimating the average physicochemical constraints acting on the gene product.
2. E-PRIME: Models *λ* as a random effect varying across phylogenetic branches and sites, allowing for the detection of episodic selection on specific properties.
3. S-PRIME: Estimates independent *λ* vectors for each codon site, resolving the fine-grained biophysical architecture of the protein.

This functional form admits a direct interpretation in the context of distance-based substitution models (Koshi and Goldstein 1998; Yang 2000; Conant et al. 2007). While the probability of fixation ($P_{\;fix}$) follows distinct scaling regimes depending on mutational fitness (Kimura 1962), modeling exchangeability as an exponential function of physicochemical distance serves as a phenomenological proxy for selection (Sella and Hirsh 2005). PRIME builds on this foundation and similar exponential parameterizations (e.g. the LCAP model of Conant et al. (2007)), but differs in three key aspects: it is a codon-based framework that separates mutational biases from selective filtering (unlike the amino-acid based model of Koshi and Goldstein 1998), resolves multiple property contributions simultaneously at the site level, and detects both property conservation and property-driven diversification. PRIME thus integrates and extends these diverse methodological efforts. We posit that the effective selection coefficient for a substitution from amino acid *x* to *y* is composed of a baseline effect $s_{0}$ (captured by $\psi^{\left(s\right)}$) and specific physicochemical costs: $s_{xy}\approx s_{0}-\sum \lambda_{i}|x_{i}-y_{i}|$. In this framework, $\lambda_{i}^{\left(s\right)}$ serves as a proxy for the intensity of the selective constraint per unit of property change.

### Amino acid properties

Models of this form can be used with different sets of amino acid properties. We consider primary properties such as chemical composition, polarity, volume, isoelectric point, and hydropathy (Conant et al. 2007), as well as composite property sets derived from dimensionality reduction techniques (Atchley et al. 2005). Within HyPhy, all property vectors are internally normalized to have zero mean and unit variance to ensure that the estimated *λ* coefficients are comparable across properties and to mitigate the impact of disparate scales on collinearity. The choice of properties represents a trade-off between interpretability and comprehensive coverage. The composite sets, referred to as “Atchley factors,” are derived from a factor analysis of 494 amino acid attributes and represent five orthogonal semantic axes: Factor 1 (Hydrophobicity), Factor 2 (Helix/Propensity), Factor 3 (Size), Factor 4 (Composition), and Factor 5 (Charge/Polarity). Primary properties, such as the Kyte-Doolittle hydrophobicity index (Kyte and Doolittle 1982) or the Zamyatnin volume scale (Zamyatnin 1972), are physically intuitive and allow selective pressures to be mapped directly to specific biochemical mechanisms. In contrast, propensities for alpha-helix or beta-sheet formation (Chou and Fasman 1974, 1979) capture marginal backbone geometric constraints. While these continuous scales do not fully account for the context-dependent nature of secondary structure formation (e.g. terminal capping or proline-induced kinks), they provide a statistically tractable proxy for the localized structural preferences that filter sequence variation.

### Hierarchical property models

We define a nested hierarchy of models, designated as 2-property through 5-property, which sequentially incorporate five primary physicochemical properties. To ensure that each primary property contributed unique information, we evaluated the Pearson correlation (*r*) between the pairwise property distance vectors for all amino acid pairs using a Mantel permutation test (50,000 permutations; online supplementary material, Table S1). Chou-Fasman alpha-helical propensity is not significantly correlated with the core packing properties: r≈−0.08 with Kyte-Doolittle hydrophobicity (p=0.33) and r≈0.14 with Zamyatnin volume (p=0.19). The only statistically significant association among the property distances is between hydrophobicity and beta-sheet propensity (r≈0.33, p=0.0027). This structural coupling is expected, as residues with high beta-sheet propensity (such as the hydrophobic, branched residues Valine, Isoleucine, and Phenylalanine) are heavily enriched in beta-sheet scaffolds to satisfy hydrogen-bonding and packing requirements in buried environments. This hierarchical approach allows us to systematically test the explanatory power of each additional property.

1. 2-property (Hydrophobicity+Volume): This baseline model includes the Kyte-Doolittle hydrophobicity index (Kyte and Doolittle 1982) and residue volume (Zamyatnin scale) (Zamyatnin 1972). These two properties represent the most fundamental constraints on protein folding: the hydrophobic effect drives the formation of the protein core, while steric constraints (volume) dictate packing density and surface accessibility. They are broadly complementary, as a residue can be small and hydrophobic (e.g. Valine) or large and hydrophobic (e.g. Tryptophan).
2. 3-property (Adding Isoelectric Point): The 3-property model adds the isoelectric point (pI), introducing charge as a distinct constraint. Electrostatic interactions are critical for protein stability, solubility, and specific ligand binding (Conant et al. 2007). While hydrophobicity governs the core, charge often dominates surface interactions and active sites, offering a dimension of constraint orthogonal to packing and burial.
3. 4-property (Adding Alpha-Helix Propensity): We extend the model by including alpha-helix propensity (Chou and Fasman 1974, 1979). This property captures local backbone geometric constraints essential for secondary structure formation. Helical propensity is distinct from simple volume or hydrophobicity, as it depends on specific side-chain branching and hydrogen bonding capabilities.
4. 5-property (Adding Beta-Sheet Propensity): The full 5-property model incorporates beta-sheet propensity (Chou and Fasman 1974, 1979), completing the description of major secondary structure determinants. This allows the model to differentiate between regions constrained by different folding motifs, providing a more comprehensive physicochemical profile of the evolutionary landscape.

These models enable a suite of systematic testing procedures. We can employ model selection criteria to determine the optimal level of complexity for a given gene or dataset; we use the small-sample corrected AICc (Sugiura 1978; Hurvich and Tsai 1989) throughout this manuscript. The application of information criteria for model selection in this context is not straightforward, for example because for model parameter estimates what constitutes the appropriate sample size is unclear (Scheffler et al. 2014). We therefore also report Bayesian Information Criterion (BIC) results for our benchmark datasets to assess the robustness of our conclusions (online supplementary material, Table S2). These criteria allow us to effectively ask how much physicochemical detail is needed to explain the observed evolution. Furthermore, Likelihood Ratio Tests (LRTs) can be used to statistically assess the significance of adding specific properties (e.g. testing 3-property vs. 2-property to isolate the importance of charge), thereby building a detailed map of the specific biochemical forces shaping protein diversity. A summary of all statistical tests and multiple testing corrections used across the different analyses in this work is presented in Table 2.

**Table 2:** Summary of statistical tests and multiple testing corrections utilized in this study.

Analysis	Statistical Test	Multiple Testing Correction	Threshold
Model Selection	AICc / BIC	N/A	Minimum IC
Global Property Constraint	LRT (1 d.f.)	N/A	p≤0.05
S-PRIME (Omnibus)	LRT (*M* d.f.)	Benjamini-Hochberg	q≤0.10
S-PRIME (Property-level)	LRT (1 d.f.)	Two-stage (Bogomolov)	α⋅R/N
Group-wise Comparisons	Mann-Whitney U/KW	Bonferroni (post hoc)	Adjusted *p*

### Incorporating branch-site heterogeneity

To capture the complexity of evolutionary processes, we employ a branch-site random effects framework ported from the BUSTED method (Murrell et al. 2015). This approach allows the importance of physicochemical properties (*λ* parameters) to vary not only across sites but also across different lineages (branches) of the phylogenetic tree. By modeling the distribution of *λ* as a random effect, we can detect episodic selection on properties—instances where specific physicochemical constraints are relaxed or intensified on particular branches. This is important for identifying transient evolutionary events, such as adaptation to a new host or immune escape, which may not be evident under models that assume constant preferences across the entire phylogeny. E-PRIME provides a flexible and robust means to infer the dynamic landscape of physicochemical constraints in protein evolution.

### PRIME testing procedure for sites

To resolve the biophysical drivers of evolution at single-residue resolution, we developed S-PRIME, a model that estimates and tests site-level importance weights ($\lambda_{i}^{\left(s\right)}$). Analogous to the Fixed Effects Likelihood (FEL) method for dN/dS (Kosakovsky Pond and Frost 2005), we assume that physicochemical constraints are temporally constant across the phylogeny at a given site but are modeled as independent parameters between sites. This site-by-site inference proceeds in two stages to balance computational efficiency with statistical precision. First, we estimate global nuisance parameters—including relative branch lengths, GTR nucleotide substitution rates, and equilibrium frequencies—jointly across the entire alignment under the standard MG94 model. These parameters are subsequently fixed to reduce the dimensionality of the site-level optimization problem, with site-level branch lengths modeled as a scalar multiple ($\alpha^{\left(s\right)}$) of the relative global lengths. In the second stage, we independently fit the site-specific parameters: the synonymous rate multiplier (*α*), the baseline log nonsynonymous/synonymous rate ratio ($\psi^{\left(s\right)}$)—as opposed to $\omega^{\left(s\right)}$—and the vector of property importance coefficients ($\lambda_{i}^{\left(s\right)}$) for each site *s*.

Statistical significance is assessed through a nested likelihood ratio test (LRT) framework. At each site *s*, we perform a set of M+1 hypothesis tests, where *M* is the number of modeled properties (e.g. M=5 for the 5-property model):

1. Global Property Test: We first evaluate the omnibus null hypothesis that evolution at site *s* is independent of all modeled physicochemical properties (i.e. $\lambda_{1}^{\left(s\right)}=\lambda_{2}^{\left(s\right)}=\cdots =\lambda_{M}^{\left(s\right)}=0$). This is an LRT with *M* degrees of freedom.
2. Individual Property Tests: If the omnibus test rejects the null, we proceed to dissect the specific drivers by testing each property *i* individually. The null hypothesis $\lambda_{i}^{\left(s\right)}=0$ is assessed using a 1-degree-of-freedom LRT, determining whether property *i* contributes a statistically significant improvement to the model fit.

**Figure 1:**
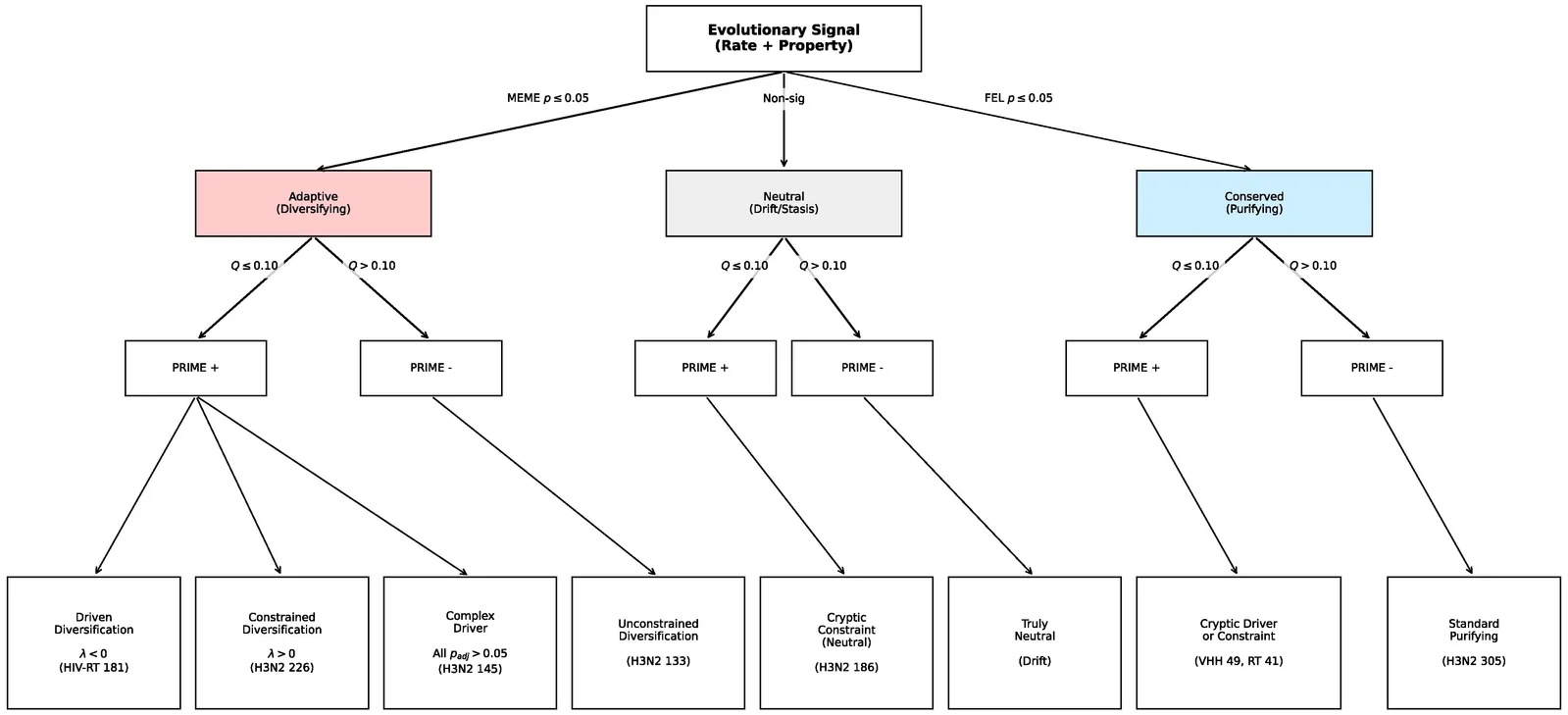
Site Classification Decision Tree. A framework for interpreting site-specific evolutionary signals by integrating substitution rate ratios (dN/dS) with physicochemical property weights (*λ*). This approach distinguishes between unconstrained diversifying selection and targeted biophysical adaptation, while also identifying cryptic constraints in neutral (dN/dS≈1) or purifying sites. Note: A site may be identified as significant by the PRIME omnibus test (indicating property dependence) even if no single property exhibits a strong individual weight, typically reflecting complex or weak constraints.

Given the large number of tests (*N* sites ×(M+1) properties), and the expectations that many or most sites could have null results, stringent multiple testing correction is essential. We employ the hierarchical procedure described by Benjamini and Bogomolov (2014) to control the selective False Discovery Rate (sFDR). This two-stage approach first applies the Benjamini-Hochberg procedure to the *N* Global Property Tests (e.g. at FDR ≤0.05) to identify sites with significant biophysical structure. Only within this subset of *R* significant sites do we evaluate the Individual Property Tests. To account for selection bias (the cherry-picking of interesting sites), we apply the Holm-Bonferroni method at an adjusted significance level of α⋅R/N. This ensures strong control of the Family-Wise Error Rate (FWER) within each selected site while maintaining global FDR control, effectively mitigating the risk of false mechanistic attributions. By combining site-level significance from both codon-based rates (FEL/MEME) and S-PRIME property tests, we classify each site into distinct biophysical selection modes according to a decision tree (Fig. 1).

### Reference models

We use three established methods as references. Each method models the nonsynonymous/synonymous rate ratio (ω=dN/dS) under different assumptions of selection heterogeneity:

1. **Fixed Effects Likelihood (FEL):** Estimates a single site-specific *ω* ratio across all branches of the phylogeny (Kosakovsky Pond and Frost 2005). It is used to identify residues under persistent purifying (ω<1) or diversifying (ω>1) selection.
2. **Mixed Effects Model of Evolution (MEME):** Allows site-specific *ω* values to vary across branches at a given site using a random-effects mixture model (Murrell, Wertheim, Moola et al. 2012). This approach provides the sensitivity to detect episodic diversifying selection that may only affect a subset of lineages.
3. **Branch-site Unrestricted Statistical Test for Episodic Diversification (BUSTED):** Provides a gene-wide test for episodic selection by modeling the distribution of *ω* ratios across all sites and branches simultaneously (Murrell et al. 2015).

### Processing datasets for large-scale structural mapping and analysis

To map evolutionary constraints to physical structure, we integrated site-level S-PRIME results with 3-dimensional protein coordinates at two complementary scales: (1) a controlled analysis of the 21 benchmark datasets (online supplementary material, Table S4), and (2) a genome-wide mapping across the mammalian proteome (Hecker and Hiller 2019).

For the benchmark datasets, we mapped alignments to available experimental structures. High-resolution coordinates (resolution ≤3.5 Å) were obtained from the Protein Data Bank (PDB). Columns of the multiple-sequence alignment (MSA) were mapped to PDB residues by performing a local sequence alignment between the consensus sequence of the MSA and the corresponding PDB chain using the Smith-Waterman algorithm (Biopython PairwiseAligner, mode = “local”). This protocol established a precise residue-to-residue mapping while correctly handling gaps and partial structural fragments.

For the genome-wide mammalian screen, we developed an automated pipeline to retrieve and map high-quality human structures for the 18,944 mammalian genes. We translated the human (hg38) coding sequence for each gene and queried the RCSB PDB using sequence search. Candidates were filtered to include only structures from *Homo sapiens* determined by X-ray diffraction or electron microscopy (excluding NMR ensembles to ensure consistent solvent accessibility calculations). We required a sequence identity threshold of ≥90% and an alignment length of ≥100 residues. When multiple structures met these criteria, we selected the best representative PDB ID and polymer entity chain by ranking them by E-value (ascending), sequence identity (descending), and alignment length (descending). The matching chain was identified and aligned to the hg38 reference sequence using the Smith-Waterman algorithm. This pipeline successfully mapped 5,125 mammalian genes to high-quality structures, encompassing 1,447,182 residues.

For both datasets, we extracted structural features from the multimeric biological assemblies (complexes) rather than isolated monomeric chains. This approach helps avoid misclassifying interface-buried residues as solvent-exposed. Solvent accessibility was computed via the Shrake-Rupley algorithm (Shrake and Rupley 1973) and normalized to Relative Solvent Accessibility (RSA) using empirical maximum solvent-accessible surface area (SASA) values (Tien et al. 2013). Residues were partitioned into three structural environments: Buried (RSA<0.05), representing the fully shielded hydrophobic core of the fold (Tien et al. 2013); Exposed (RSA≥0.25), representing solvent-exposed surface residues with sufficient area to participate in molecular interactions (Adamczak et al. 2004); and Intermediate (0.05≤RSA<0.25), capturing partially buried residues lining crevices and binding pockets (Ma and Wang 2015). Secondary structure assignments were generated using the DSSP algorithm (Kabsch and Sander 1983) or extracted from MMCIF dictionaries. To characterize the local packing environment, we calculated the contact number within a 10 Å radius ($CN_{10}$, defined by C*α*–C*α* distances).

### Comparison with protein language models

To investigate the relationship between explicit physicochemical constraints and the implicit representations learned by deep learning, we benchmarked site-specific PRIME importance weights (*λ*) against residue embeddings from the ESM-2 protein language model (Lin et al. 2023). For each dataset, we computed per-residue embeddings using the esm2_t33_650M_UR50D architecture (layer 33, 1280 dimensions) for the primary reference sequence. A critical methodological step involved the precise alignment of these residue-level embeddings ($L_{seq}$) with the alignment-level PRIME inferences ($L_{aln}$). We mapped each nongap residue in the reference to its corresponding MSA column, filtering the results to include only those sites present in the reference structure. To distill the high-dimensional PLM representation into interpretable components, we performed Principal Component Analysis (PCA) on the aligned ESM embeddings for each dataset. We then quantified the alignment between the two frameworks by calculating the Spearman rank correlation (*ρ*) between site-specific PRIME property weights and the top principal components of the ESM-2 embeddings. Following Rives et al. (2021), who established that the primary axes of PLM latent spaces frequently track fundamental biophysical features, we defined the recoverability of a property as the maximum absolute correlation between that property’s weight and any of the top five principal components. This protocol accounts for the high-dimensional and nonorthogonal nature of PLM embeddings, where biophysical signal is often distributed across multiple latent axes. By mapping each property to the most relevant semantic axis within the primary informative subspace (the top five PCs typically capture a large fraction of total variance), we ensure that our recoverability metric remains robust to the specific orientation of the latent space. This approach formally tests whether the black-box predictions of the PLM are capturing the same underlying biophysical signal as the explicit PRIME model, providing a quantitative bridge between statistical phylogenetics and representation learning.

### Genome-wide screening protocol

To characterize the landscape of physicochemical constraints across the mammalian proteome, we analyzed a high-quality subset of coding sequences from the mammalian gene alignment datasets (Hecker and Hiller 2019). The initial collection was filtered to ensure robust phylogenetic inference, retaining only alignments that met the following criteria: (1) contained at least 10 sequences; (2) had a coding length of at least 50 codons; and (3) exhibited a total tree length between 0.5 and 50 substitutions per site. This procedure yielded a final dataset of 18,944 gene alignments. For each gene, we fitted a suite of evolutionary models: the standard MG94 baseline, the CoRa model (Weber and Whelan 2019), and the G-PRIME and E-PRIME implementations of the 5-property PRIME model. To provide rate-based evolutionary context, we utilized the three reference models, FEL, MEME, and BUSTED, mentioned in the previous section. All model fits were performed using HyPhy v2.5.94 or later. We employed the 5-property parameterization (Hydrophobicity, Volume, Isoelectric Point, Alpha-Helix Propensity, Beta-Sheet Propensity) as the default for this screen, based on its superior performance in our preliminary benchmarks. Statistical significance of property conservation or diversification was assessed using Likelihood Ratio Tests (LRTs) corrected for multiple testing via the Benjamini-Hochberg (BH) procedure (FDR≤0.05). Genes where model fitting failed to converge for any of the comparison models were excluded from the final comparative analysis to ensure a matched dataset.

### Simulation strategy for S-PRIME performance assessment

To systematically evaluate the statistical performance of S-PRIME in detecting site-specific physicochemical constraints, we designed a simulation framework that models the realistic scenario of localized biophysical pressure against a background of neutral evolution. Our approach assesses both the control of False Positive Rate (FPR) and the power to detect true positive signals under varying regimes of constraint intensity. For each of the 24 benchmark datasets (online supplementary material, Table S4), we first estimated global model parameters under the neutral null hypothesis (MG94), obtaining gene-specific estimates for branch lengths, nucleotide substitution biases (*θ*), and codon equilibrium frequencies (*π*). These parameters served as the seed for generating replicate alignments under two distinct conditions. First, to establish a baseline for Type I error control, we simulated alignments entirely under the null MG94 model (scenario null_mg94), where all property importance weights are strictly constrained to zero (λ=0). Second, to characterize detection power, we implemented a chimeric simulation strategy, where we defined a set of over 20 selective scenarios representing diverse biophysical regimes (detailed in online supplementary material, Table S3). These include Conserved and Diversifying scenarios where a single focal property is constrained (e.g. $\lambda_{\mathrm{Vol}}=3.0$) while all other parameters are set to zero to ensure a clean background. We also included Empirical scenarios derived from clusters of site-level constraints observed in biological data, and Easy/Hard multiproperty combinations. For each replicate, we generated two parallel alignments: a Null alignment evolved under MG94 and an Alternative alignment evolved under the 5-property G-PRIME model parameterized with the scenario-specific *λ* vector. We then constructed a spliced dataset by retaining the first 80% of sites from the Null alignment and appending the final 20% of sites from the Alternative alignment. This protocol injects a specific biophysical signal into a defined genomic window, allowing us to strictly quantify the method’s sensitivity to detect localized constraints while maintaining the phylogenetic context of the original gene. Power was calculated using the proportion of Alternative sites correctly identified as significant by the S-PRIME omnibus test after False Discovery Rate (FDR) correction (q≤0.10 using the Benjamini-Hochberg procedure):

### Robustness to model misspecification

We evaluate model performance under conditions that violate its generative assumptions using the Pyvolve package (Spielman and Wilke 2015). Specifically, we leverage the Mutation-Selection (MutSel) framework (Halpern and Bruno 1998) to generate sequence data where selective pressures are biophysically informed but mathematically distinct from the exponential rate-multiplier form used by PRIME. We define site-specific fitness profiles using a Gaussian Stabilizing Selection model: $F(aa)=-k\cdot (P_{aa}-T_{0}{)}^{2}$ where *P* is the amino acid property value and $T_{0}$ is the biophysical optimum. This independent generative process allows us to test if PRIME recovers meaningful physicochemical signals when the true model is related but not identical to the inference framework. We perform inference using the 3-property and 5-property S-PRIME models and assess significance using the 2-stage Bogomolov-Benjamini procedure (q≤0.1).

We utilize the empirical phylogeny of the Vertebrate Rhodopsin (RH1) benchmark dataset (N=38 taxa) for these simulations. To ensure sufficient mutational depth for site-level resolution, we scale the tree by a factor of 3.0, resulting in a total tree length of approximately 11.28 substitutions per site. We designed the multi-scenario property recovery (MSPR) framework to systematically evaluate stability and specificity across 7 regimes (S1-S7; 50 replicates each). Each replicate consists of 400 sites, partitioned into distinct selective and control windows. Control windows consist of sites evolved under a neutral MG94 model (ω=1.0) with the same background mutation parameters as the selective partitions, providing a paired internal baseline for each replicate. To selective windows, we apply a nonuniform mutation background (HKY with κ=2.5) to force the model to separate mutational bias from selective filtering. The 7 regimes are:

1. **S1 (Sensitivity Gradient):** We partition sites into four 100-site windows and calibrate the selection coefficient (*k*) to target specific levels of variation, measured by the Effective Number of Amino Acids ($N_{eff}=e^{H}$, where the entropy *H* is computed over the stationary amino-acid frequency distribution). We simulate Strong ($N_{eff}=3.0$), Medium ($N_{eff}=6.0$), and Weak ($N_{eff}=10.0$) constraints, with the final 100 sites serving as a neutral control.
2. **S2 (Property Specificity):** We partition sites into four 100-site windows representing independent selection on three distinct properties—Hydrophobicity, Volume, and Isoelectric Point—to verify that PRIME correctly assigns significance to the intended biophysical axis. The final 100 sites served as a neutral control.
3. **S3–S5 (Compositional Robustness):** We test if S-PRIME is misled by sites that exhibit compositional bias toward certain amino acids (e.g. highly hydrophobic sites) in the absence of selective dynamics. For each primary property (S3: Hydrophobicity, S4: Volume, S5: Isoelectric Point), we generate two 150-site partitions with identical stationary distributions (*π*) but different dynamics: a MutSel partition (true rate suppression) and a Frequency-Biased Neutral (FBN) partition. We evolve FBN sites under an MG94 model where *ω* is fixed at 1.0, and we explicitly set the codon equilibrium frequencies to match the stationary distribution of the corresponding selective MutSel model. This protocol ensures that both partitions have the same amino acid composition, isolating the effect of substitution rate dynamics from frequency bias. The remaining 100 sites serve as a neutral control.
4. **S6 (Heterotachy Stress Test):** We evaluate whether unmodeled rate variation across lineages confounds property inference by evolving 200 sites under a strict episodic regime: neutral evolution (ω=1.0) across the majority of the tree, punctuated by purifying selection (ω=0.05) localized to a specific terminal clade. The remaining 200 sites evolve neutrally across the entire phylogeny.
5. **S7 (Interpolation of Unmodeled Constraints):** We investigate model behavior when the true selective sieve is absent from its explicit parameterization. We simulate selection for Aromaticity (a strict preference for Phenylalanine, Tyrosine, and Tryptophan), alongside a “nonaromatic” partition favoring all other amino acids as a negative control. This scenario tests whether the framework mathematically projects unmodeled constraints onto the closest available biophysical proxies.

### Comparison with experimental fitness landscapes

To validate the biological relevance of inferred physicochemical constraints, we benchmarked S-PRIME against Deep Mutational Scanning (DMS) data for the H3N2 Hemagglutinin protein (Lee et al. 2018). We integrated the A/Perth/16/2009 (H3N2) sequence used in the DMS assay into a historical H3N2 alignment (Bush et al. 1999) and performed a masked imputation analysis as follows. For each site in the Perth H3N2 sequence, we discarded the observed codon and inferred the conditional probability distribution of all 61 sense codons given the phylogenetic history and the fitted model parameters using S-PRIME. This procedure parallels the masked language modeling objective used in deep learning.

For comparison, we generated zero-shot predictions using the ESM-2 protein language model (esm2_t33_650M_UR50D) (Lin et al. 2023). We masked each position in the translated Perth H3N2 amino acid sequence and extracted the probability distribution over the 20 standard amino acids. We evaluated the alignment between model predictions and experimental fitness using three metrics calculated over variable sites: (1) Pearson correlation, capturing the linear relationship between predicted probabilities and experimental preferences; (2) Spearman rank correlation, assessing the monotonicity of the ranking; and (3) Jensen-Shannon Divergence (JSD), quantifying the information-theoretic distance between the predicted and experimental distributions. Additionally, we defined a strongly preferred set for each site (residues with probability >0.10) and calculated the Jaccard index and total probability mass coverage to assess the model’s ability to identify the subset of viable amino acids.

## Results

### Empirical benchmarking of G-PRIME and E-PRIME models

To evaluate G-PRIME and its branch-site extension (E-PRIME), we benchmarked them on 21 diverse alignments and 3 specialized datasets (Lucaci et al. 2023) (online supplementary material, Table S4): Cytochrome C Oxidase I (COXI, membrane), Type I Collagen (COL1A1, fibrous), and Amelogenin (AMELX, disordered). We compared these against the standard MG94×REV baseline and CoRa (Weber and Whelan 2019) (Table 3). Note that our focus is on biophysically interpretable models; while empirical codon models (Kosiol et al. 2007) can improve fit, they add up to 190 parameters without direct biophysical interpretability, whereas G-PRIME adds only *D* parameters (one per property) to Muse-Gaut models.

**Table 3:** Architectural summary and parameterization of evaluated evolutionary models.

Model	Heterogeneity	Added Params	Suggested Application
MG94×REV	None	0	Baseline/Null model
G-PRIME	Global $\lambda_{i},\psi$	*D*	Genome-wide constraints
E-PRIME	Random effects (*K* classes)	2(K−1)+KD	Episodic adaptation
S-PRIME	Fixed effects (per site)	≈L(D+1)	Site-specific constraints

PRIME configurations are distinguished by the level of selection heterogeneity they capture, ranging from genome-wide averages to site-specific biophysical drivers. We report the nature of heterogeneity, the number of additional parameters relative to the MG94+REV baseline (*D*: number of physicochemical properties; *K*: number of mixture components; *L*: number of alignment sites), and the intended biological applications for each model.

**Figure 2:**
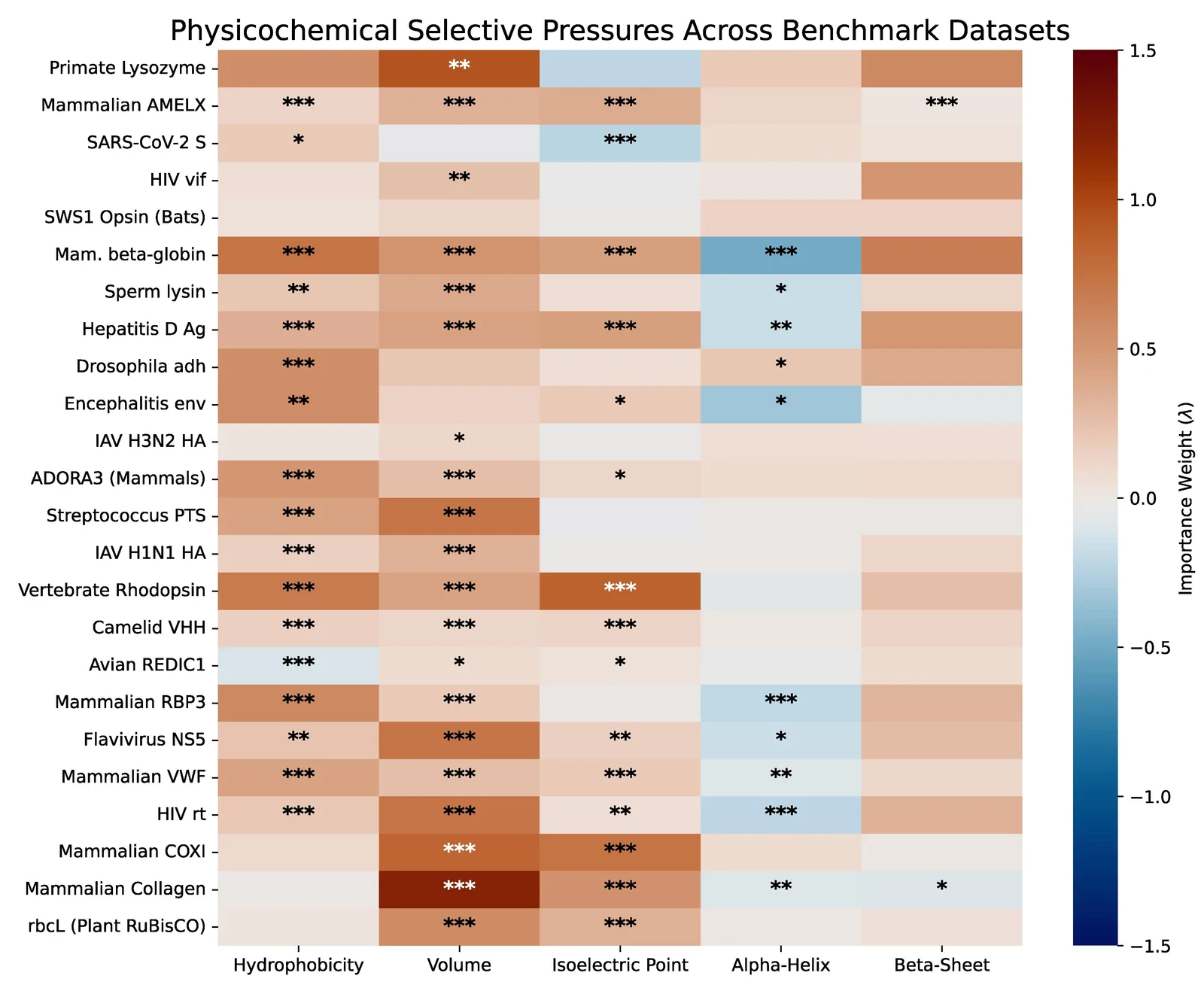
Physicochemical selective fingerprints across 24 benchmark datasets. The heatmap displays estimated property importance weights (*λ*) for the 5-property G-PRIME model. Red cells indicate property conservation (purifying selection), while blue cells indicate a drive for property change (diversifying selection). Asterisks denote *λ* values significantly different from zero (Likelihood Ratio Test; * p<0.05, ** p<0.01, *** p<0.001). Datasets are ordered by total evolutionary information (L×T). Hydrophobicity and volume emerge as nearly universal constraints, while secondary structure propensities and isoelectric point serve as the primary substrates for adaptive tuning.

Explicitly modeling properties yields consistent fit improvements, with G-PRIME and E-PRIME statistically rejecting the MG94 baseline in Likelihood Ratio Tests (p≤0.05) and remaining preferred under both AICc and BIC (online supplementary material, Table S2). The magnitude of fit improvement (ΔAICc) scales with total evolutionary information (L×T); expected substitutions correlate strongly with ΔAICc (Spearman ρ≈0.65,p<0.001). Large, divergent datasets (e.g. rbcL and Mammalian Collagen) exhibit massive gains (ΔAICc>990). Consequently, the 5-property G-PRIME model serves as the robust default when mutational depth is sufficient. Beyond overall fit, G-PRIME characterizes the specific properties driving selection. Global importance weights (*λ*) across the benchmark (Fig. 2; raw values in online supplementary material, Table S5) reveal the predominant constraint modes: positive values (red) denote property conservation (purifying selection)—with hydrophobicity and volume acting as nearly universal constraints—whereas negative values (blue) highlight primary substrates for diversifying selection and adaptive tuning (specifically secondary structure propensities and isoelectric point).

Comparing E-PRIME against a 2-component BUSTED baseline confirms that modeling variable physicochemical constraints outperforms the assumption of uniform amino acid exchangeability. E-PRIME significantly improves fit over BUSTED (Table 4)—exceeding 1800 AICc units for rbcL and 1200 units for Mammalian Collagen—demonstrating that selective pressures vary not just in rate (dN/dS), but in the biophysical rules governing amino acid exchanges. For example, in Vertebrate Rhodopsin, adding hydrophobicity and volume (2-property E-PRIME) improves fit over BUSTED by ΔAICc≈375, while adding isoelectric point (3-property) yields a further jump (ΔAICc≈632), supporting the electrostatics of spectral tuning. Similarly, Hepatitis D Antigen benefits from isoelectric point modeling ($\Delta {\text{AICc}}_{3-2}\approx 145$), reflecting charge constraints in RNA-binding, while Mammalian Collagen fit is maximized by the 5-property E-PRIME (ΔAICc≈1231), consistent with the rigid volume packing of its triple helix.

**Table 4:** Comparison of E-PRIME models against the BUSTED baseline for 24 benchmark alignments.

Dataset	$\pi_{m}$	2-prop	3-prop	4-prop	5-prop	Atchley	*I*
Primate Lysozyme	0.9	21.4 (0.8)	23.0 (0.8)	18.9 (0.9)	34.5 (0.8)	14.6 (1.0)	−10.9
Mammalian AMELX	0.7	12.7 (0.8)	29.4 (0.9)	30.0 (0.8)	33.8 (1.0)	40.5 (1.0)	−15.5
SARS-CoV-2 S	1.0	−2.4 (1.0)	15.1 (1.0)	15.4 (1.0)	19.0 (1.0)	11.3 (1.0)	−2.3
HIV vif	0.9	21.4 (0.8)	29.5 (0.8)	23.2 (0.9)	40.5 (0.9)	49.3 (0.9)	−4.5
SWS1 Opsin (Bats)	0.8	0.7 (0.7)	26.6 (0.8)	14.3 (0.7)	43.9 (0.8)	13.0 (0.7)	−37.9
Mam. *β*-globin	0.9	84.9 (0.8)	−31.1 (1.0)	209.5 (0.9)	253.5 (0.9)	157.3 (0.9)	−38.6
Sperm lysin	0.9	62.1 (0.9)	52.2 (0.8)	90.6 (0.9)	77.0 (0.8)	13.2 (0.8)	−19.5
Hepatitis D Ag	1.0	148.3 (0.9)	293.6 (0.9)	291.1 (0.9)	330.6 (0.9)	235.7 (0.9)	−48.4
Drosophila adh	1.0	96.1 (1.0)	102.9 (0.9)	103.5 (0.9)	107.9 (0.9)	82.6 (1.0)	−35.1
Encephalitis env	1.0	7.9 (0.5)	16.7 (0.7)	22.6 (0.6)	19.9 (0.6)	14.3 (0.9)	−9.6
IAV H3N2 HA	1.0	24.5 (0.9)	57.3 (1.0)	34.9 (0.9)	83.0 (1.0)	54.1 (1.0)	−79.5
ADORA3 (Mammals)	0.8	109.0 (0.7)	115.3 (0.8)	143.9 (0.7)	144.0 (0.7)	65.9 (0.9)	−41.6
Streptococcus PTS	0.9	185.2 (0.8)	218.9 (0.9)	216.7 (0.9)	214.2 (0.9)	72.7 (0.8)	−77.9
IAV H1N1 HA	1.0	133.5 (0.9)	147.4 (0.9)	165.3 (1.0)	154.8 (0.9)	83.2 (1.0)	−5.8
Vertebrate Rhodopsin	1.0	375.4 (0.9)	631.8 (1.0)	654.0 (1.0)	681.4 (1.0)	331.7 (0.9)	−124.1
Camelid VHH	0.9	−22.9 (0.7)	0.3 (0.7)	5.9 (0.7)	134.3 (0.9)	171.3 (0.9)	1.6
Avian REDIC1	1.0	11.5 (1.0)	−1.0 (0.6)	18.5 (1.0)	−3.7 (0.5)	−0.2 (1.0)	21.5
Mammalian RBP3	0.9	319.7 (0.9)	331.4 (0.9)	450.9 (0.8)	503.4 (0.8)	189.0 (0.8)	−131.0
Flavivirus NS5	1.0	156.4 (0.9)	175.9 (0.9)	223.2 (0.9)	218.4 (0.9)	104.3 (0.9)	−62.7
Mammalian VWF	0.9	279.4 (0.9)	344.3 (0.7)	425.8 (0.8)	358.5 (0.7)	203.1 (0.8)	−32.6
HIV rt	1.0	454.6 (1.0)	520.7 (1.0)	473.7 (1.0)	719.0 (1.0)	261.7 (1.0)	−284.5
Mammalian COXI	0.8	107.3 (1.0)	160.2 (1.0)	166.4 (1.0)	170.0 (1.0)	64.1 (1.0)	−46.1
Mammalian Collagen	0.9	698.4 (1.0)	950.1 (0.9)	1176.8 (1.0)	1231.0 (1.0)	822.5 (1.0)	−238.1
rbcL (Plant RuBisCO)	1.0	1105.6 (1.0)	1817.5 (1.0)	1816.9 (1.0)	1823.0 (1.0)	559.8 (1.0)	−495.3

We report *Δ*AICc relative to BUSTED, with the weight of the dominant mixture component ($\pi_{m}$) provided in parentheses for E-PRIME models and in the second column for BUSTED. Hierarchical property models are defined as: 2-property (Hydrophobicity, Volume), 3-property (+ Isoelectric Point), 4-property (+ Alpha-Helix Propensity), and 5-property (+ Beta-Sheet Propensity). Datasets are sorted by L×T. The Interaction Information (*I*) column quantifies synergy (I<0) using the 5-property model. Best-fitting models (*Δ*AICc >0) are highlighted in bold.

The inclusion of alpha-helix propensity (4-property) also yields significant fit improvements across multiple datasets, such as Mammalian *β*-globin ($\Delta {\text{AICc}}_{4-3}\approx 240$) and Mammalian RBP3 ($\Delta {\text{AICc}}_{4-3}\approx 120$). To address whether the Chou-Fasman alpha-helical propensity scale acts as a proxy for other unmodeled forces, we systematically evaluated the Mantel correlation (*r*) between the pairwise amino acid distance matrix of the Chou-Fasman scale (CHOP780201) and those of all other 566 indices in the AAindex1 database (Kawashima et al. 2008). Beyond expected strong distance-level correlations with other independently derived helical propensity scales (e.g. r=0.95 with Palau et al., 1981; r=0.92 with Robson-Suzuki, 1976), the scale correlates only weakly to moderately with general physicochemical properties. The strongest strictly nonstructural correlates include Fauchere’s steric parameter (r=0.32,p=0.014), Fauchere’s carboxyl $pK_{a}$ (r=0.28,p=0.028), and O’Neil-DeGrado peptide stability ΔG in urea (r=0.26,p=0.036), while correlation with primary packing parameters remains very low: r≈−0.08 with Kyte-Doolittle hydrophobicity (p=0.35) and r≈0.12 with Bigelow residue volume (p=0.25). This distance-based Mantel scan supports that alpha-helical propensity captures a distinct mode of selective constraint that cannot be reduced to simple packing or charge properties.

The mixture components recovered by E-PRIME (online supplementary material, Table S6) partition evolutionary history into distinct selective regimes. The dominant component (typically >70% weight) exhibits strict conservation of volume and hydrophobicity, reflecting core structural constraints. The minor component captures protein-specific adaptations: diversifying selection on surface charge or hydrophobicity in viral antigens (SARS-CoV-2 Spike, IAV HA), or relaxed purifying constraints in metabolic enzymes (rbcL, HIV-1 RT). Finally, we evaluated whether rate heterogeneity (BUSTED) and property constraints (G-PRIME) are synergistic. Using the Interaction Information metric, $I=\Delta {\text{AICc}}_{G-\mathrm{PRIME}}+\Delta {\text{AICc}}_{\mathrm{BUSTED}}-\Delta {\text{AICc}}_{E-\mathrm{PRIME}}$, we observed widespread synergy (I<0) across the benchmark—including rbcL (I≈−495) and Mammalian Collagen (I≈−238). This negative interaction suggests that rate and property models capture complementary dimensions: sites often appear rate-conserved likely because they are filtered through a specific biophysical sieve.

### Genome-wide profile of physicochemical selection in mammalian genes

To evaluate the broader applicability of our framework, we deployed G-PRIME and E-PRIME to characterize the physicochemical landscape of the mammalian proteome. We analyzed a high-quality subset of 18,944 mammalian genes (alignments with ≥10 sequences, ≥50 codons, tree length 0.5–50, and successful fits across all 5 comparison models: MG94, CoRa, G-PRIME, E-PRIME, and BUSTED). Given that the 5-property model provided the optimal fit for the majority of benchmark datasets (Tables S2 and 4), we restricted this large-scale analysis to the G-PRIME and E-PRIME 5-property models. We formulated three primary biophysical expectations: (1) pervasive conservation of hydrophobicity and volume to maintain the hydrophobic core and prevent steric clashes (Baseline Expectation) (Richards 1974; Dill 1990); (2) stricter conservation of beta-sheet propensity than alpha-helix propensity, reflecting the rigid nature of beta-sheet scaffolds (Structural Signal); and (3) targeting of alpha-helix propensity for diversifying selection (λ<0) or neutrality, consistent with the tuning of helical stability in flexible linkers and disordered regions to modulate binding affinity (Regulatory Mode) (Zarin et al. 2019).

**Figure 3:**
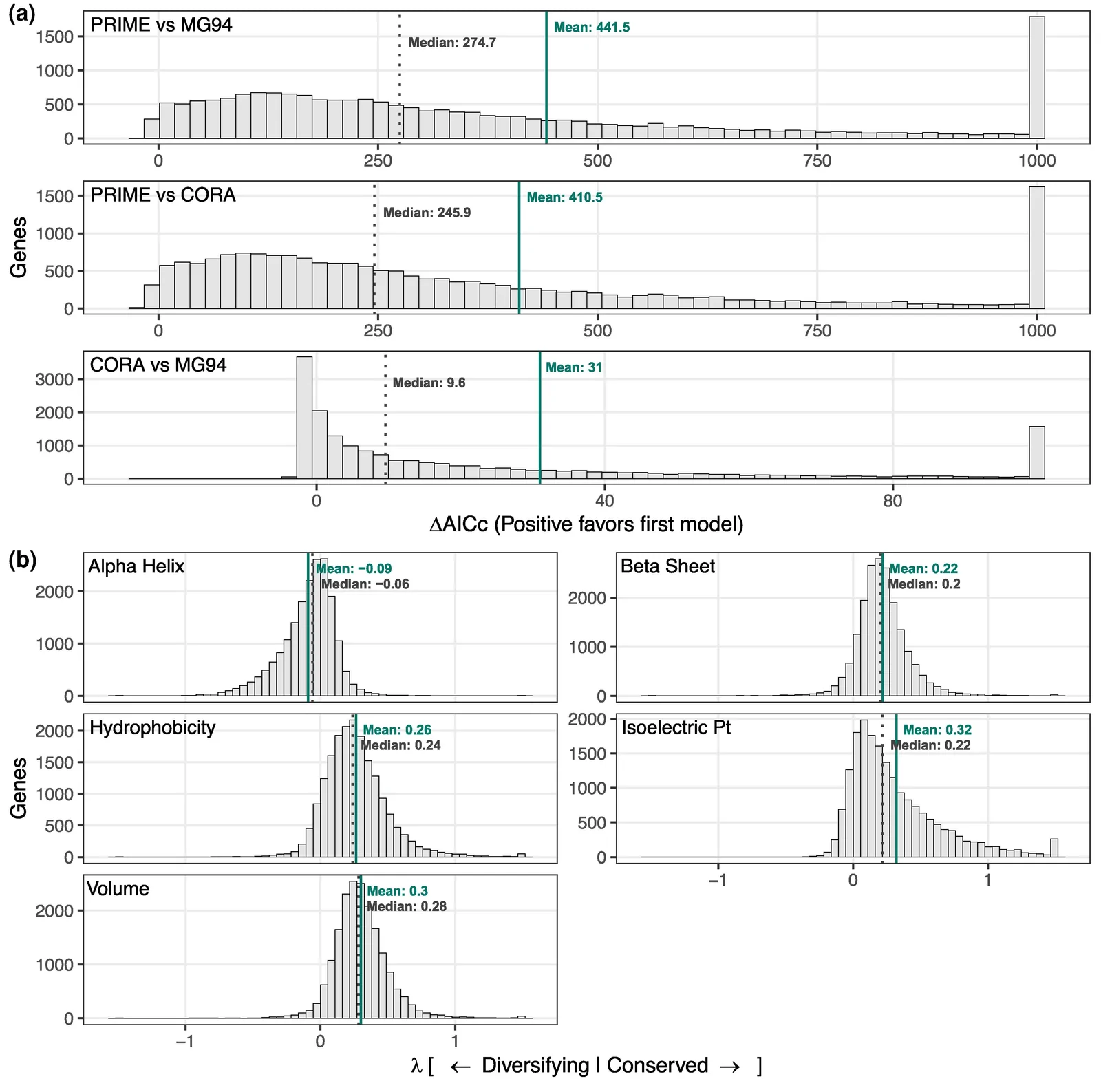
Genome-wide analysis of physicochemical selection in 18,944 mammalian genes. a) Model selection performance showing distributions of *Δ*AICc for three comparisons: Global 5-property G-PRIME vs. MG94 baseline, G-PRIME vs. CoRa, and CoRa vs. MG94. Histograms are faceted by comparison with independent scales; values are clamped at −25 and +1,000 for visibility. b) Distributions of global property importance weights (*λ*) across the proteome. Positive *λ* values signify property conservation (purifying selection), while negative values signify a drive for physicochemical change (diversifying selection). Data are clamped at ±1.5 for visualization. Summary statistics (mean, median, and 95% intervals) for each distribution are provided in the inset boxes.

**Figure 4:**
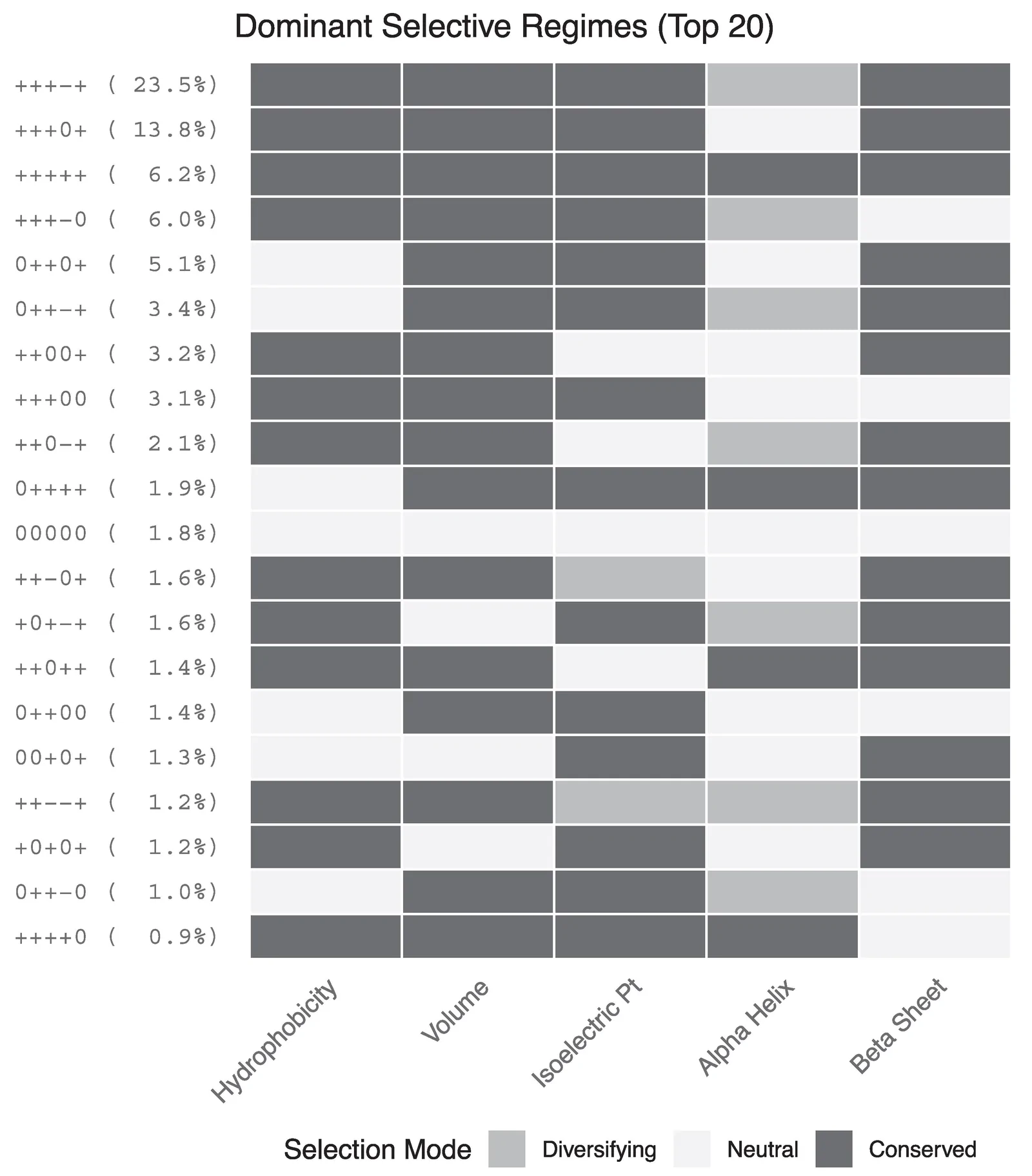
Dominant Physicochemical Selective Regimes. The top 20 most frequent combinations of property constraints across 18,944 mammalian genes. Properties are ordered as: Hydrophobicity, Volume, Isoelectric Point, Alpha-Helix, Beta-Sheet. For each property, genes were classified as Conserved (dark gray), Changing (light gray), or Neutral (white) based on an FDR threshold of 0.05. The patterns are ordered by frequency, with the percentage of the proteome shown in parentheses. The most common regime involves broad conservation coupled with selection for changing alpha-helix propensity.

Our analysis of 18,944 mammalian genes provides support for these biophysical principles. Consistent with the Baseline Expectation, core properties are inferred to be under pervasive purifying selection: Hydrophobicity ($\bar{\lambda }\approx 0.265$) and Volume ($\bar{\lambda }\approx 0.301$) predominantly exhibit positive weights (Fig. 3b), statistically significant (FDR ≤0.05 via LRT) in 74.2% and 85.8% of the proteome, respectively. In agreement with the Structural Signal, beta-sheet propensity is widely conserved ($\bar{\lambda }>0$; significant in 75.5% of genes). In contrast, in agreement with the Regulatory Mode, alpha-helix propensity is inferred to have a uniquely broad distribution centered near zero ($\bar{\lambda }\approx -0.092$), with a substantial fraction of the proteome showing neutrality or active selection for change (44.0% with λ<0). Classifying genes by their combinations of property-specific selective regimes (FDR ≤0.05; Fig. 4) confirms that selection for change is remarkably sparse and targeted, almost exclusively affecting alpha-helix propensity (e.g. the most common regime, 23.5% of genes, features broad conservation coupled with selection for changing alpha-helix propensity). Multiproperty diversification regimes are rare but biologically coherent; for example, the ++−−+ regime (1.2% of genes), combining core structural conservation with selection for changing isoelectric point and alpha-helix propensity, is strongly enriched for host defense enzymes such as Serine Peptidases (GO:0008236, $p_{adj}=1.6\times 10^{-11}$; e.g. *KLK8*, *ELANE*) and glycosyltransferases (GO:0008417, $p_{adj}=6.5\times 10^{-6}$; e.g. *FUT3*, *FUT6*), reflecting rapid adaptive remodeling of surface interfaces under host-pathogen co-evolution.

**Figure 5:**
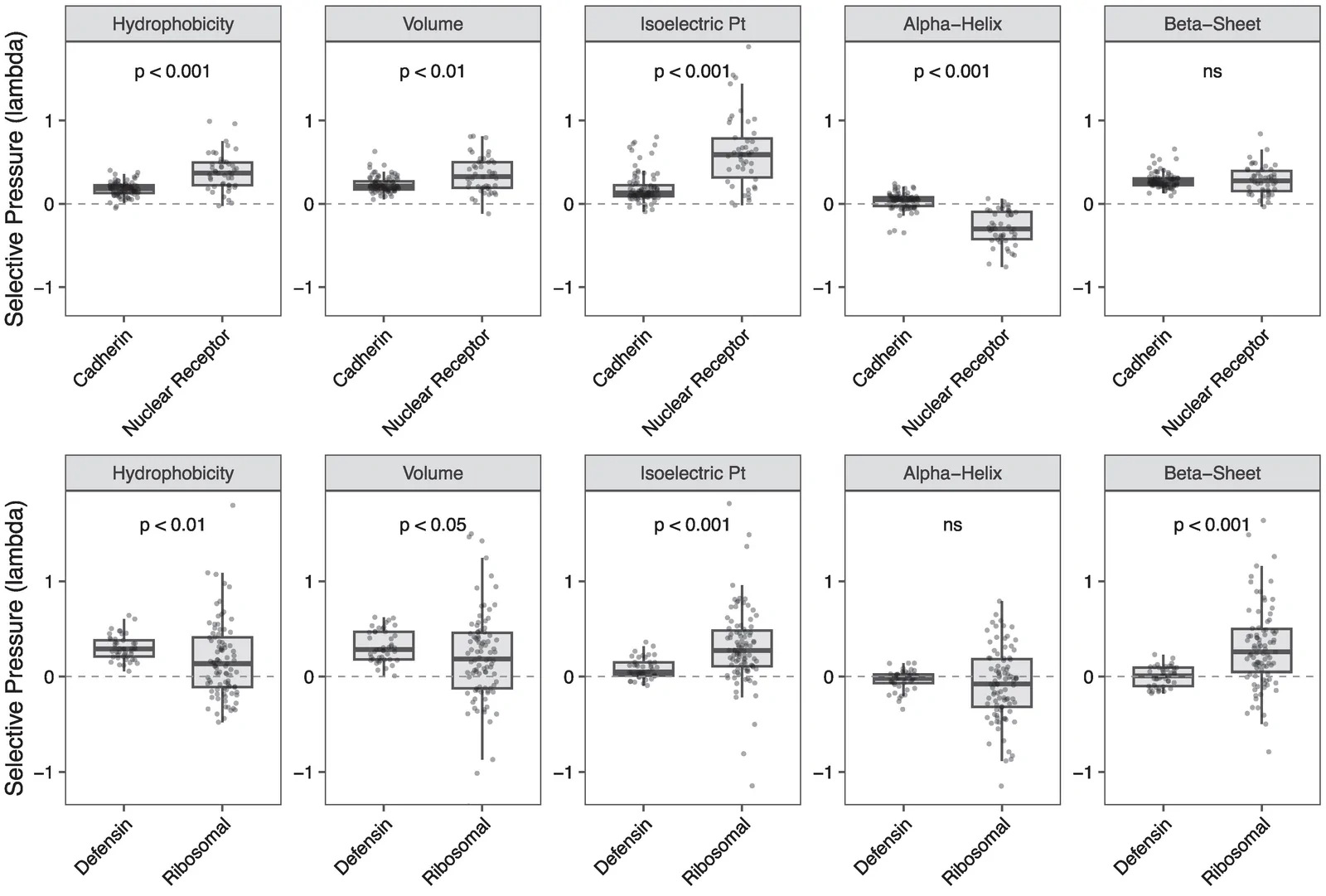
G-PRIME distinguishes structural, regulatory, and interaction-driven constraints. Top: Comparison of global property importance weights (*λ*) between Cadherins and Nuclear Receptors. While both families conserve beta-sheet propensity (structural stability), Nuclear Receptors show strong diversifying selection on alpha-helix propensity (regulatory tuning), contrasting with the neutral evolution in Cadherins. Bottom: Comparison between Defensins and Ribosomal proteins. Ribosomal proteins exhibit strict conservation of isoelectric point and beta-sheet propensity (RNA-binding and machine assembly), while Defensins show significantly reduced constraint on these properties, consistent with rapid adaptive evolution in immune defense. P-values derived from Mann-Whitney U test are shown above each comparison. Boxes represent the interquartile range (IQR), horizontal lines denote the median, and whiskers extend to the most extreme data points within 1.5 × IQR of the box. Individual data points are shown as jittered points.

To validate that G-PRIME can infer biologically meaningful selective signals, we performed two case studies contrasting protein families with divergent structural and functional paradigms (Fig. 5). For this and other group-wise comparisons, we utilized the nonparametric Mann-Whitney U test (for pairwise comparisons) and Kruskal-Wallis test (for multigroup analysis), as they do not assume normality and are robust to the skewed distributions of evolutionary parameters. These case studies utilize standard biological sample sizes (N≤95 genes per family) where nonparametric rank tests provide robust statistical inference. First, comparing Cadherins (N=92; rigid adhesion scaffolds) against Nuclear Receptors (N=46; dynamic regulatory switches) supports that while both conserve beta-sheet propensity ($\bar{\lambda }\approx 0.28$), 76% of Nuclear Receptors likely undergo significant diversifying selection on alpha-helix propensity (λ<0), whereas Cadherins were likely subjected to weak conservation or neutrality ($\bar{\lambda }\approx 0.03$). This corresponds to a Common Language Effect Size (CLES) of 0.938, meaning that a randomly selected Cadherin has a 93.8% probability of having a higher/more conserving *λ* weight than a randomly selected Nuclear Receptor; Mann-Whitney U test, $p=5.7\times 10^{-17}$). We also observed statistically significant differences in other properties: Hydrophobicity (CLES = 0.818, $p=1.3\times 10^{-9}$), Isoelectric Point (CLES = 0.822, $p=7.8\times 10^{-10}$), and Volume (CLES = 0.665, $p=1.6\times 10^{-3}$), whereas beta-sheet propensity did not differ significantly (CLES = 0.502, p=0.97). Second, comparing Defensins (N=45) against Ribosomal Proteins (N=95) supports that Ribosomal proteins strictly conserve isoelectric point ($\bar{\lambda }\approx 0.41$; significant in 54% of genes), likely facilitating RNA binding, whereas Defensins show relaxed charge constraint ($\bar{\lambda }\approx 0.08$; 53% neutral), permitting charge drift to recognize diverse pathogen surfaces. This difference corresponds to a CLES of 0.764 ($p=5.1\times 10^{-7}$). Similarly, Ribosomal proteins exhibit greater conservation of beta-sheet propensity (CLES = 0.783, $p=7.1\times 10^{-8}$) and lower conservation of hydrophobicity (CLES = 0.347, $p=3.5\times 10^{-3}$) and volume (CLES = 0.392, $p=4.0\times 10^{-2}$), while alpha-helix propensity did not differ significantly (CLES = 0.449, p=0.33).

The 5-property G-PRIME model consistently outperforms alternative parameterizations across the proteome. While the CoRa model improves upon the MG94 baseline in 76.5% of datasets, the G-PRIME model offers a far more substantial advance, providing a superior fit for 98.6% of analyzed genes (Fig. 3a). Furthermore, G-PRIME outperformed CoRa itself in 98.5% of cases. This strong support indicates that the property-specific, quantitative modeling of physicochemical constraints provides a far more accurate description of the evolutionary process than rate-based or binary classification approaches. We further quantified the relationship between rate heterogeneity (captured by BUSTED) and physicochemical heterogeneity (captured by G-PRIME) by computing the Interaction Information (*I*) across the genome. While the addition of rate heterogeneity alone (BUSTED vs. MG94) yielded a mean improvement of 211.1 AICc units, and G-PRIME alone (G-PRIME vs. MG94) yielded 441.5 units, the combined E-PRIME model provided a mean gain of 723.6 units over the MG94 baseline. Consistent with our benchmark findings, we observed pervasive synergy between these dimensions: the mean interaction information was −71.0, with 84.4% of genes exhibiting synergy (I<0). This result suggests that for the vast majority of the mammalian proteome, accounting for both temporal rate variation and property-specific physicochemical constraints yields a model fit superior to the sum of their individual contributions, suggesting that these frameworks capture orthogonal and synergistic components of the evolutionary process.

**Figure 6:**
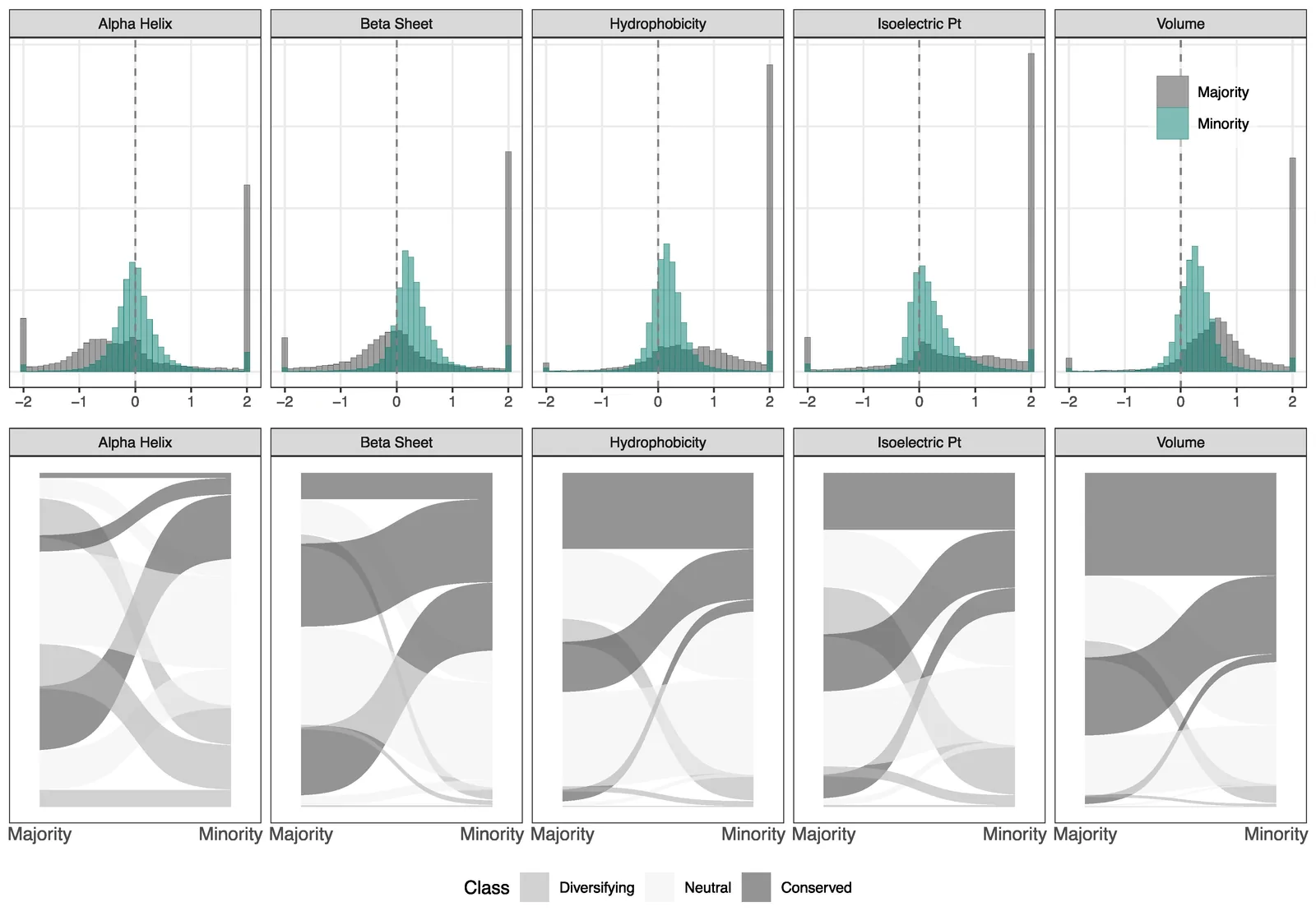
Divergent Selective Regimes in the Mammalian Proteome. Top: Density distributions of property importance weights (*λ*) for the Majority (dark grey) and Minority (teal) mixture components of the E-PRIME model. The Minority component is characterized by intense conservation of Beta-sheet propensity, Volume, and Hydrophobicity, while Isoelectric Point and Alpha-Helix propensity show greater variance. Bottom: Alluvial plot tracking the flow of evolutionary states (Conserved, Neutral, Diversifying) from the Majority to the Minority component (FDR≤0.05). Note the pronounced shift in Beta-sheet propensity from neutral/diversifying in the Majority to conserved in the Minority (scaffold regime), and the increased diversifying selection on Isoelectric Point in the Minority regime.

Analyzing the parameter estimates from the E-PRIME mixture components (Fig. 6) reveals that constraints are rarely static; the average gene exhibits a secondary evolutionary regime spanning 18.5% of its history. Hydrophobicity and volume remain rigid (only <3% of genes fluctuate between conserved and diversifying modes), whereas surface electrostatics and secondary structure elements serve as the primary substrates for episodic adaptation. A prominent scaffold signature (diversifying beta-sheet propensity in the majority component, but strict conservation in the minority; $\lambda_{maj}<0,\lambda_{min}>0$) is observed in 20.3% of genes (e.g. 86% of the PLEKHG family), indicating critical structural preservation of beta-strand elements. Conversely, the charge tuning signature ($\lambda_{maj}>0,\lambda_{min}<0$) is consistent with functional adaptation where global electrostatic stability is maintained while specific interfaces adapt, a mode highly enriched in Olfactory Receptors (GO:0004984, $p_{adj}<10^{-20}$), Fucosyltransferases (64%), and NLRP sensors (57%). Finally, rare regimes suggest the occurrence of active evolutionary conflicts: the double diversification of charge ($\lambda_{maj}<0,\lambda_{min}<0$) is enriched in antiviral factors like *APOBEC3* (30%) and *SLFN* (67%), while cryptic diversification ($\lambda_{maj}\approx 0,\lambda_{min}<0$) is a hallmark of immune receptors (e.g. *KIR*, *LILRA*, Defensins; GO:0019730, $p_{adj}\approx 4\times 10^{-5}$). Discordant selection on packing constraints (hydrophobicity conserved in one component but diversifying in the other) is enriched in Zinc Finger transcription factors (GO:0006357, $p_{adj}\approx 0.01$), reflecting their modular alternation between rigid DNA-binding domains and flexible transactivation regions. These results indicate that while the hydrophobic core remains rigid, surface electrostatics and secondary structure elements serve as the primary substrates for episodic adaptation.

### Site-level physicochemical selection in viral and vertebrate genes

While aggregate gene-wide models capture broad selective patterns, they average over the heterogeneous biophysical requirements of individual residues. Here, we deploy the S-PRIME inference framework to resolve selective forces at single-residue resolution. By estimating a vector of property-specific weights (*λ*) for each site, S-PRIME moves beyond the phenomenological detection of rate variation to explicitly identify *which* physicochemical properties are constrained or diversified. We apply this high-resolution lens to (1) uncover cryptic constraints in sites that appear neutral by standard rate-based metrics, (2) map the structural anatomy of physicochemical selection, and (3) dissect the biophysical logic of key adaptations in viral and vertebrate proteins.

To illustrate how S-PRIME partitions evolutionary signals into specific biophysical mechanisms, we analyzed the evolution of Influenza A H3N2 Hemagglutinin (HA). As the primary surface antigen, HA is subject to intense selective pressure to evade host immunity while maintaining receptor binding and membrane fusion. Standard methods (dN/dS) effectively identify sites under positive selection (Yang et al. 2000; Murrell, Wertheim, Moodley et al. 2012), such as those within the canonical antigenic epitopes. However, these estimates remain agnostic to the biophysical constraints that channel these adaptive shifts. Consider Site 226, located within the receptor binding pocket of the HA head domain. In our H3N2 benchmark dataset, this site exhibits a clear signature of diversifying selection (dN/dS≈22, FEL $p<10^{-10}$). This elevated dN/dS is driven by Glutamine-to-Leucine (Q226L) substitutions, which shift viral specificity from avian-type to human-type sialic acids (Rogers and Paulson 1983). While dN/dS correctly flags this position as rapidly evolving, it treats the Q:L substitution as an abstract token exchange. In contrast, S-PRIME analysis offers a more detailed explanation of this functional switch, classifying it as an instance of constrained diversification. The model identifies significant diversifying selection on Hydrophobicity ($\lambda =-2.49,p_{\mathrm{adj}}<10^{-11}$) and Volume ($\lambda =-1.81,p_{\mathrm{adj}}<10^{-5}$), capturing the chemical transition from polar Glutamine to a larger, hydrophobic Leucine. This adaptive shift occurs against a backdrop of strict conservation for Isoelectric Point ($\lambda =15.0,p_{\mathrm{adj}}=0.0004$) and Alpha-Helix Propensity ($\lambda =11.8,p_{\mathrm{adj}}=0.001$). Structural mapping (PDB: 4FNK) reveals the biophysical basis for this complexity: Site 226 is largely buried within the receptor binding pocket (RSA=0.06) and forms part of a beta-bridge. This environment necessitates strict preservation of the local electrostatic and packing architecture, channeling adaptation rather than admitting broad variation. A different exampled is offered at Antigenic Epitope A Site 145, which is located in an intermediate solvent-accessible environment (RSA=0.14, Beta-strand). The S-PRIME omnibus test identifies pronounced property-dependence ($Q=3.6\times 10^{-11}$), yet under strict Holm-Bonferroni correction, no single dimension emerges as the sole driver. This identifies Site 145 as a complex driver, where selection likely acts on a distributed combination of surface properties rather than a single axis. This distinguishes it from sites like Site 186 (Epitope B), which appears neutral to dN/dS tests ($p_{\mathrm{MEME}}=0.06$), but which S-PRIME resolves as a cryptic constraint driven by the conservation of Isoelectric Point ($\lambda =13.3,p_{\mathrm{adj}}<0.001$). By partitioning selection into these interpretable channels, S-PRIME achieves a level of mechanistic resolution that was previously unattainable with standard codon models.

Across the entire H3N2 HA gene (N=329), S-PRIME identified 12 sites with significant physicochemical constraints (FDR ≤0.10). These results provide a complementary view of adaptation to standard methods: only five sites were flagged by both S-PRIME and MEME (p<0.05). S-PRIME exclusively identified 7 sites missed by MEME—including Site 186 and Site 310 (Stem)—revealing cryptic biophysical signals where substitution turnover is permitted only among chemically similar residues. Conversely, MEME identified 12 sites not detected by S-PRIME, which can be explained as cases of physicochemically unconstrained diversification. Of these 12 sites, 42% are fully solvent-exposed, including Antigenic Site A positions 133 and 135. Structural analysis confirms that these unconstrained sites lack secondary structure (Coil), allowing immune evasion to proceed through a permissive range of substitutions without disrupting the protein fold. This discrepancy reflects a distinction in methodology: while dN/dS methods identify *where* and *when* selection acts by estimating substitution rates, S-PRIME distinguishes targeted structural shifts from biochemically unconstrained evolution by characterizing *how* the biophysical nature of selection differs between them.

Applying this classification framework across the benchmark datasets (N=9,998 sites) resolves 576 sites (5.8%) with significant biophysical structure (S-PRIME FDR ≤0.10; online supplementary material, Table S7). Among residues identified as being under diversifying selection (MEME p≤0.05), nearly half (45.9%; 180/392) is associated with a detectable biophysical structure. Of these structured adaptive sites, 58.9% resolved as constrained diversification, where adaptation occurs against a backdrop of significant conservation for at least one property. The remaining adaptive sites were classified as Complex Drivers (28.3%) or driven diversification (12.8%), where selection favors property change without detectable structural rigidities. Within the neutral class, 4.3% of sites (226/5,300) exhibited significant structure, identifying them as Cryptic Constraints. Finally, of the 4,306 sites identified as evolving under purifying selection, significant physicochemical constraints were resolved for 170 (3.9%). While the majority of these represent Cryptic Constraints (strict property conservation), we also infer instances of Cryptic Drivers (0.7% of all sites), where specific properties are driven to change despite global purifying selection. online supplementary material, Figure S1 visualizes these conditional probabilities, revealing the distinct biophysical fingerprints found across the selection spectrum.

Our classification framework can distinguish biophysically driven selection from relaxed purifying selection or neutral drift. For example, in Mammalian RBP3—a specialized lipocalin where relaxed selection dominates vision-reduced lineages—MEME identifies four sites (0.97%) under episodic positive selection (p≤0.05). S-PRIME classifies three of these (75%) as Unconstrained Diversification (elevated rate without biophysical structure), with only a single site (0.24%) showing biophysically directed selection. Similarly, in the structurally constrained Mammalian Collagen triple helix, MEME flags 49 sites (3.4%) under positive selection. S-PRIME resolves 61% of these (30 sites) as Unconstrained Diversification, leaving only 19 sites (1.3%) with biophysically driven or constrained adaptation. This sparse footprint is consistent with localized adaptations (e.g. to body mass or aquatic lifestyles) within a highly conserved structural scaffold.

To illustrate the biophysical interpretation of S-PRIME parameters under drug-induced selection, we examined significant sites in HIV-1 Reverse Transcriptase (RT). S-PRIME identifies catalytic residue 184 (located in the conserved YMDD motif of the active site) as undergoing purifying selection on hydrophobicity ($\lambda_{\mathrm{Hydro}}=15.0,q<10^{-6}$) despite a high rate of substitution (46 events). The observed mutations (M→V, M→I) involve minimal change in hydrophobicity, preserving the active site core while conferring resistance to lamivudine (3TC) and emtricitabine (FTC). The model correctly infers that while residue identity can change, the biophysical property—hydrophobic packing—must be strictly maintained. Similarly, residue 100 (a key site for NNRTI resistance) exhibits strong volume conservation ($\lambda_{\mathrm{Vol}}=15.0,q<0.001$), reflecting steric constraints in the NNRTI binding pocket that limit viable amino acids to those maintaining specific spatial occupancy. In contrast, surface-exposed residue 214 exhibits a signature of diversifying selection on isoelectric point ($\lambda_{\mathrm{pI}}\approx -10.9,q=0.02$), where electrostatic remodeling may facilitate immune evasion or solubility adaptation without disrupting the protein fold.

Finally, S-PRIME resolves adaptation in Vertebrate Rhodopsin (RH1), where standard dN/dS-based methods have historically failed to identify the molecular basis of spectral tuning. Experimental ancestral reconstruction previously identified 12 amino acid sites responsible for spectral shifts (Yokoyama et al. 2008), yet standard dN/dS analyses in PAML did not identify these positions as under positive selection. S-PRIME analysis identifies significant biophysical selection (q≤0.10) at five of these critical tuning sites (Sites 83, 122, 261, 289, and 292). At Site 292, for instance, which is associated with spectral tuning, dN/dS analyses yield a nonsignificant result (p=0.49), whereas S-PRIME reports a biophysical constraint driven by isoelectric point and volume (q<0.001). This demonstrates that S-PRIME can uncover functional adaptations that are missed by traditional rate-based approaches.

A logistic regression analysis (detailed in online Supplementary material) reveals that S-PRIME significance is driven by substitution depth (redundancy ratio *R*) rather than simple rate acceleration, and is optimized for detecting specific “toggling” behaviors, as shown in Section “Simulation-based performance and robustness of S-PRIME”. Furthermore, at the site level, S-PRIME property constraints are globally decoupled from rate-based selection coefficients (dN/dS); however, at significant positive selection sites, rapid adaptation along one biophysical axis is typically channeled against a background of purifying conservation on other properties (online supplementary material, Figure S2).

### Decoding latent structural semantics in protein language models

To investigate whether the explicit physicochemical constraints modeled by S-PRIME capture the same fundamental biological structure as implicit deep learning representations, we compared site-specific property weights (*λ*) against residue embeddings from the ESM-2 protein language model (Lin et al. 2023). For each of the benchmark datasets, we computed per-residue embeddings for the primary reference sequence and performed Principal Component Analysis (PCA) to distill the high-dimensional PLM representation into its dominant axes of variation. We then quantified the alignment between the two frameworks by calculating the Spearman rank correlation (*ρ*) between the S-PRIME importance weights and the top principal components of the aligned ESM-2 latent space.

Confirming this hypothesis, we observed statistically significant, albeit modest, correlations across the benchmark datasets (mean max |ρ|≈0.19, max ≈0.34). To control for the multiple comparisons inherent in selecting the maximum correlation across five principal components per property, we applied a Bonferroni correction (adjusted significance threshold of α=0.01); all reported correlations remain highly significant (p<0.001, adjusted) owing to the large number of sites per alignment, indicating that selecting the maximum correlation is controlled. This correspondence is particularly notable given the fundamental differences between the two frameworks: S-PRIME infers constraints from a single alignment using an explicit biophysical model, whereas ESM-2 learns statistical patterns from an extensive universe of unrelated sequences. While the relatively low magnitude of these correlations suggests that ESM-2 captures higher-order contextual features that a marginal site-specific model misses, the fact that PRIME’s properties—specifically hydrophobicity and volume—consistently track the principal axes of the PLM latent space suggests that S-PRIME effectively recovers the core biophysical rules that partially underlie the opaque logic of deep learning. This alignment provides independent, data-driven validation that the physicochemical properties modeled here indeed represent primary semantic features governing protein evolution.

### The structural anatomy of physicochemical selection

To validate the physical reality of the inferred property selection coefficients ($\lambda_{i}^{\left(s\right)}$), we mapped S-PRIME results to three-dimensional protein structures at two complementary scales: (1) a controlled analysis of 5,938 sites across the 21 benchmark datasets with experimental structures, and (2) a genome-wide structural mapping of 1,447,182 residues across thousands of mammalian genes. For both datasets, we calculated Relative Solvent Accessibility (RSA) directly from multimeric biological assemblies to account for quaternary interface contacts, and classified residues into Buried (RSA<0.05), Intermediate (0.05≤RSA<0.25), and Exposed (RSA≥0.25) environments. These thresholds align with established structural bioinformatics conventions for partitioning residue environments: an RSA of 0.25 is the standard for defining solvent-exposed surface residues with sufficient area to mediate interactions (Adamczak et al. 2004), while the 0.05 threshold defines residues belonging to the shielded hydrophobic core (Tien et al. 2013), and the intermediate bin (0.05–0.25) captures the twilight zone of partially buried residues lining crevices and binding pockets (Ma and Wang 2015).

**Figure 7:**
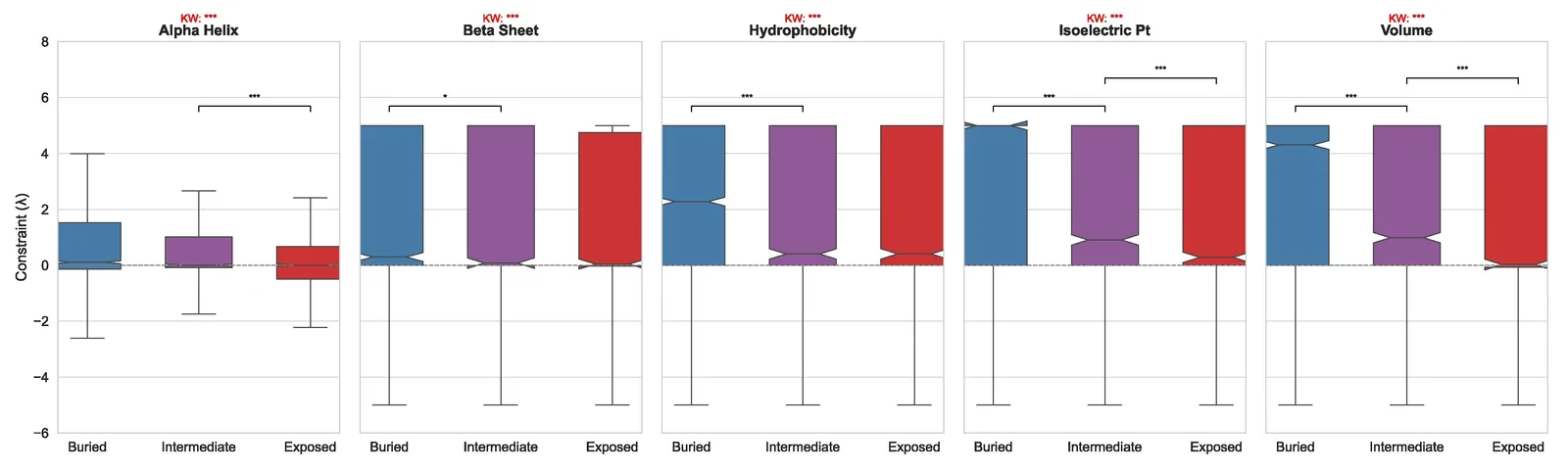
The Structural Anatomy of Selective Pressure. Distributions of site-specific property importance weights (*λ*) stratified by Relative Solvent Accessibility (RSA) classes across 21 benchmark datasets. Properties include (left to right): Alpha-helix propensity, Beta-sheet propensity, Hydrophobicity, Isoelectric Point, and Volume. Brackets indicate significant pairwise differences (Mann-Whitney U test, Bonferroni corrected); Kruskal-Wallis (KW) significance is shown above each panel. Results demonstrate a pervasive gradient where core residues are subject to significantly more stringent physicochemical constraints than surface residues. Boxes represent the interquartile range (IQR), horizontal lines denote the median, and whiskers extend to the most extreme data points within 1.5 × IQR of the box (outliers not shown).

**Figure 8:**
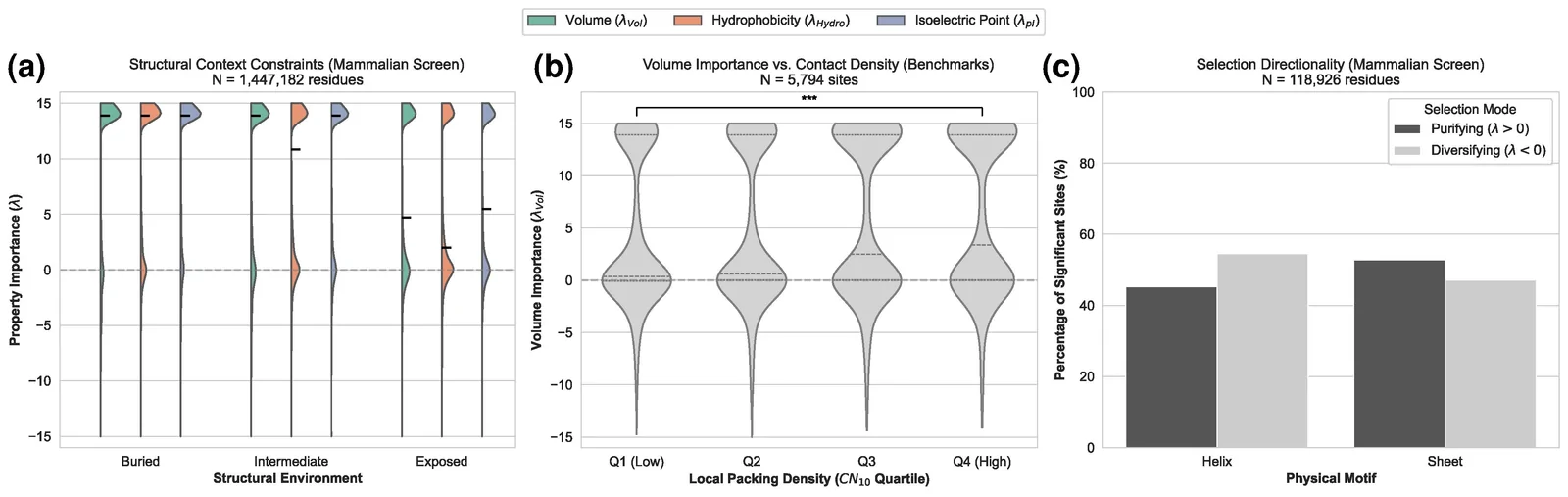
Structural Validation of PRIME Physicochemical Correlates. a) Distribution of actual (signed) property constraints (*λ*) for Volume, Hydrophobicity, and Isoelectric Point (pI) across three structural environments (Buried, Intermediate, Exposed) in the genome-wide mammalian screen (1,447,182 residues, $p<10^{-16}$, Kruskal-Wallis). Buried and Intermediate regions show heightened constraints relative to the exposed Surface. b) Positive correlation between physical contact density (coordination number within 10 Å) and volume importance ($\lambda_{\mathrm{Vol}}$) across benchmark datasets ($p<10^{-16}$). c) Proportions of significant residues in physical secondary structure motifs subject to purifying (λ>0, dark gray) vs. diversifying (λ<0, light gray) selection ($p=3.66\times 10^{-57}$, Mann-Whitney U), demonstrating that beta-sheets function predominantly under purifying conservation whereas alpha-helices are enriched for diversifying adaptation. Horizontal lines inside half-violins A) represent group medians; dashed/dotted lines inside plot B) represent quartiles and medians.

This dual-scale analysis reveals a congruent, depth-dependent gradient of biophysical constraint, demonstrating that the structural core of proteins constrains property-specific variation (Figs. 7 and 8a). Differences between structural classes were assessed using nonparametric Kruskal-Wallis and Mann-Whitney U tests to account for the non-normal nature of site-specific parameter estimates. Because of the large sample sizes, we report the Common Language Effect Size (CLES) to quantify the magnitude of these differences.

For the benchmark datasets, absolute property constraints (|λ|) are significantly elevated in the interior core and decay toward the exposed surface for Volume (CLES=0.601, $p=1.7\times 10^{-28}$), Isoelectric Point (CLES=0.596, $p=3.4\times 10^{-26}$), and Hydrophobicity (CLES=0.580, $p=1.8\times 10^{-18}$; Fig. 7). This structural gradient is recapitulated in the large-scale mammalian screen using actual (signed) property constraints (Fig. 8a, Kruskal-Wallis $p<10^{-16}$ for all properties). In the mammalian screen, volume constraints (Buried median $\lambda_{\mathrm{Vol}}=13.89$ vs. Exposed median 4.73; CLES=0.606), charge constraints (Buried median $\lambda_{\mathrm{pI}}=13.89$ vs. Exposed median 5.46; CLES=0.635), and hydrophobicity constraints (Buried median $\lambda_{\mathrm{Hydro}}=13.89$ vs. Exposed median 1.99; CLES=0.626) are all maintained in the interior to prevent packing defects and the burying of unpaired charges. We find that these volume constraints scale continuously with physical contact density—measured by coordination number within 10 Å ($CN_{10}$) across the benchmark datasets ($R\approx 0.11,p<10^{-16}$; Fig. 8b)—supporting that the biophysical selective sieve tracks local atomic packing.

While secondary structure propensities also show significant variation ($p<10^{-5}$), the magnitude of the depth-dependent effect is less severe than for core packing and electrostatics, suggesting that structural grammar is maintained throughout the fold. However, S-PRIME reveals a significant divergence in evolutionary modes between secondary structure motifs when comparing the proportion of purifying vs. diversifying selection in physical helices and physical sheets across significant mammalian sites ($p=3.66\times 10^{-57}$, Mann-Whitney U; CLES=0.531; Fig. 8C). Beta-sheets act predominantly as rigid structural scaffolds under purifying conservation (median $\lambda_{\mathrm{Sheet}}=+0.142$, with 52.8% of sites under purifying selection). Conversely, alpha-helices exhibit a strong propensity for adaptive biophysical tuning (median $\lambda_{\mathrm{Helix}}=-0.256$, with 54.5% of sites under diversifying selection), supporting that helices frequently function as the flexible substrates for evolutionary adaptation.

To investigate whether this core-to-surface constraint decay is a universal genomic rule, we calculated the Spearman rank correlation (*ρ*) between RSA and signed selection coefficients (*λ*) for each of the 3,025 mammalian genes with at least 200 structurally mapped residues. The distributions of these per-gene correlations are overwhelmingly negative across all three primary properties (mean $\rho_{\mathrm{Hydro}}=-0.191$, mean $\rho_{\mathrm{Vol}}=-0.177$, and mean $\rho_{\mathrm{pI}}=-0.226$; online supplementary material, Figure S3), indicative of constraint decay as a general biophysical program of protein evolution.

### Comparison with experimental fitness landscapes

We compared PRIME inference against Deep Mutational Scanning (DMS) data for the Influenza A H3N2 Hemagglutinin protein. We analyzed 280 variable sites in the A/Perth/16/2009 (H3N2) background, comparing the predicted amino acid preferences from S-PRIME imputation with the experimental fitness measurements. For comparison, we also evaluated zero-shot predictions from the ESM-2 protein language model. S-PRIME shows a moderate correspondence with experimental results, yielding a mean Pearson correlation of r=0.605 and a mean Spearman rank correlation of ρ=0.157 across all variable sites. In comparison, ESM-2 achieved a lower Pearson correlation (r=0.533) but a higher Spearman correlation (ρ=0.305). The mean Jensen-Shannon Divergence (JSD) for S-PRIME was 0.382, while ESM-2 exhibited a lower divergence (JSD=0.327). We observed that while the Pearson correlation is moderately high (r≈0.61), the Spearman rank correlation is notably lower (ρ≈0.16). This discrepancy is associated with the S-PRIME model assigning high probability (>90%) to a single amino acid, while experimental DMS measurements show a more varied distribution of preferences across the sub-optimal amino acid states. Because the relative ranks of these minor states in S-PRIME are frequently dominated by low-level transition-rate noise, the rank-based Spearman coefficient is heavily penalized despite strong agreement on the primary preferred residues (online supplementary material, Figure S4). These results suggest that while global representations from large-scale protein language models better support the ranking and information-theoretic structure of the fitness landscape, PRIME’s explicit biophysical model provides a competitive linear correspondence and superior mechanistic interpretability.

To further assess the model’s ability to identify the most viable residues, we defined a strongly preferred set for each site (amino acids with predicted or experimental probability >0.10). S-PRIME achieved a mean Jaccard index of 0.501, comparable to ESM-2 (0.460). Furthermore, the residues in the PRIME preferred set capture, on average, 21.2% of the experimental preference mass, a value slightly lower than ESM-2 (21.9%). While this coverage is limited, it demonstrates that the biophysical constraints inferred from evolutionary history align with a meaningful subset of the experimentally determined viable state space, performing comparably to state-of-the-art predictive frameworks.

**Figure 9:**
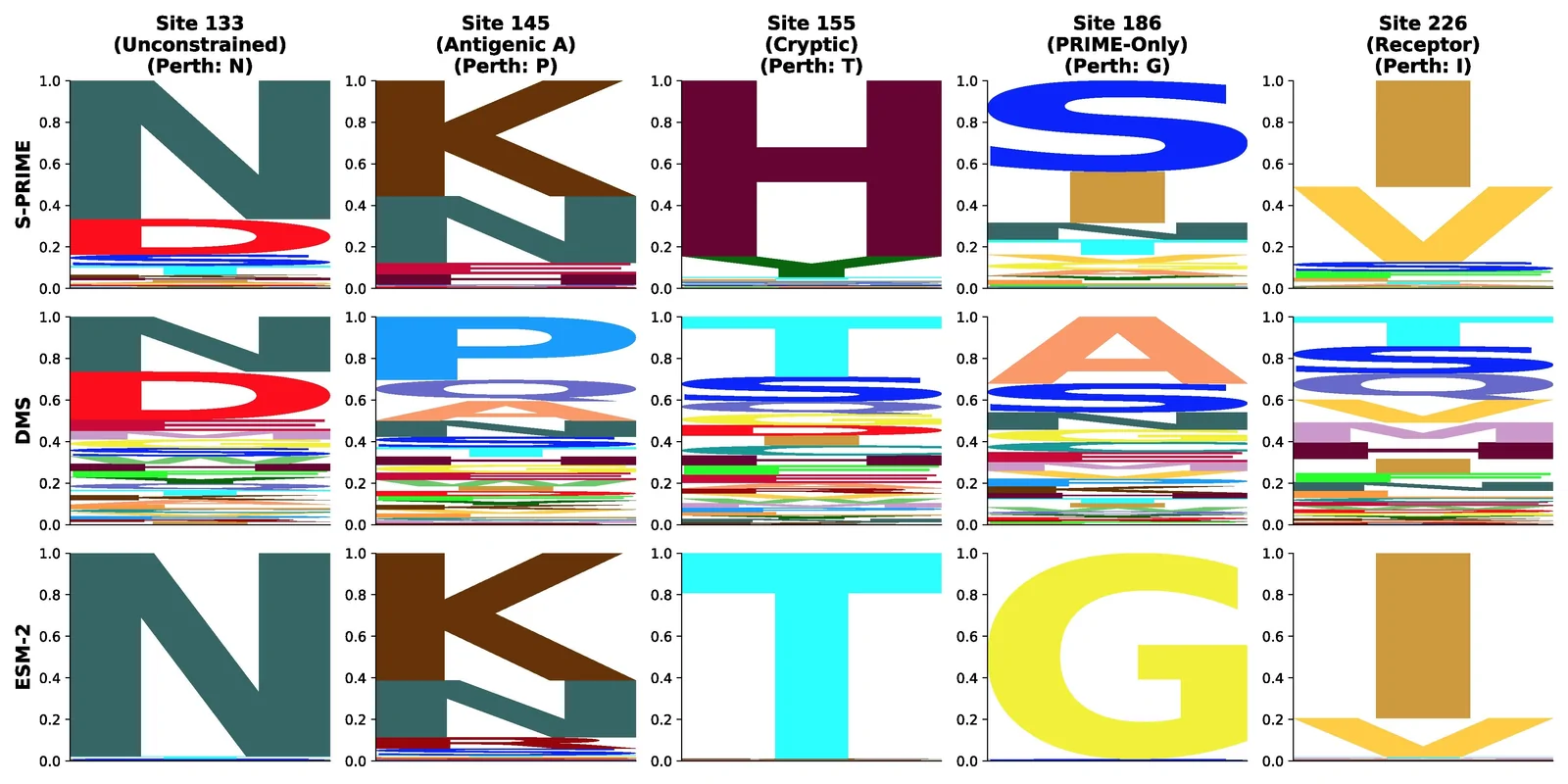
Validation against Experimental Fitness Landscapes. Side-by-side comparison of amino acid preferences for five key sites in H3N2 Hemagglutinin, as determined by S-PRIME phylogenetic imputation (top row), experimental Deep Mutational Scanning (DMS, middle row), and zero-shot predictions from the ESM-2 protein language model (bottom row). Letter height represents relative probability or preference. S-PRIME effectively identifies the subset of chemically viable residues, capturing both strict conservation (e.g. Site 186) and targeted biophysical diversification (e.g. Site 226), performing comparably to the large-scale ESM-2 framework.

A site-specific dissection of several significant biophysical drivers (online supplementary material, Table S8) reveals that PRIME identifies residues where specific properties appear to influence viability. For example, Site 186 (Epitope B), which appears neutral by traditional rate-based metrics, is resolved as a cryptic constraint driven by the conservation of Isoelectric Point (λ=13.3). DMS results show a somewhat restricted state space at this position, with preferences broadly aligning with chemically similar residues (e.g. ASN, GLY, CYS). Similarly, Site 226 exhibits diversifying selection on Hydrophobicity and Volume, reflecting the biophysical transition required for host-receptor adaptation. These patterns are visually corroborated by the side-by-side comparison of S-PRIME, DMS, and ESM-2 logos (Fig. 9). Collectively, these results suggest that PRIME recovers aspects of the biophysical rules governing protein fitness, providing a useful surrogate for interpreting experimental landscapes from phylogenetic history.

### Simulation-based performance and robustness of S-PRIME

We assessed the statistical performance of S-PRIME through a comprehensive simulation study encompassing approximately 5,000 replicate alignments across 21 selective scenarios. The aggregate statistics for false positive rate and power across all genes and scenarios are detailed in online supplementary material, Table S9. Analysis of the neutral baseline (scenario null_mg94) demonstrates strong calibration of the omnibus test (online supplementary material, Figure S5A). We find that the test is generally conservative: across 69,774 variable neutral sites, the raw False Positive Rate (FPR) at α=0.05 is only 1.9%, significantly below the nominal threshold. This conservative behavior arises because the 5-property model is overparameterized for typical sites with low informational depth, leading to a p-value distribution that is stochastically larger than uniform. Following Benjamini-Hochberg correction (q≤0.10), the mean FPR across all datasets is effectively 0.00%, confirming that the framework is highly specific and robust to stochastic noise under neutral evolution. Detection power is strongly stratified by informational content, with data-rich alignments achieving high sensitivity (online supplementary material, Figure S5B).

**Figure 10:**
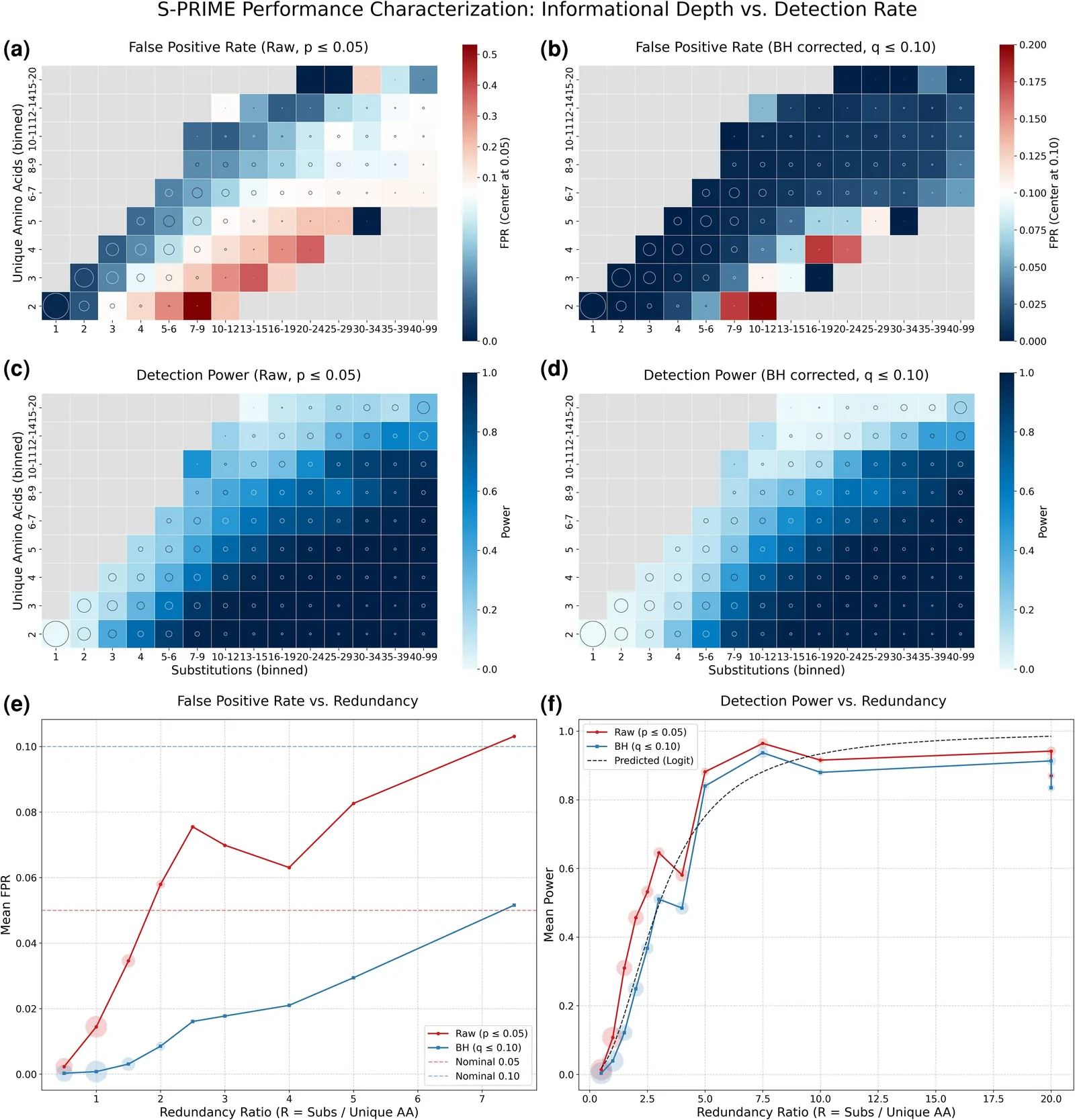
Informational determinants of S-PRIME performance. a–d) Binned heatmaps illustrating the relationship between the number of nonsynonymous substitutions, amino acid diversity, and detection rates. FPR (Panels a, b) is evaluated on the Null partition (λ=0), while Power (Panels c, d) is evaluated on the Alternative partition. Panels A and C show results for the raw omnibus test (p≤0.05), while b and d show results after Benjamini-Hochberg correction (q≤0.10). Heatmap cells represent mean rates across all replicates; cell area (adaptive bubble strokes) denotes the relative number of simulation sites in that bin. Cells with no simulation data are colored gray. e, f) Pooled performance as a function of the redundancy ratio (*R*). Bubble area in E and F is proportional to the total number of sites in each *R* bin.

Detection power is primarily governed by the informational redundancy of substitutions at a site rather than global tree length alone (Fig. 10c–f). We identify the redundancy ratio ($R=N_{subs}/N_{aa}$) as the fundamental measure of detection, defining the practical boundaries of biophysical discovery. Formal logistic regression across ∼200,000 simulation sites (alternative model) confirms that *R* is a highly effective predictor of detection sensitivity (AUC=0.91). Power scales sharply and nonlinearly with *R*: predicted probability of detection is low (7.9%) at sites with R=1.0, but increases to 28.5% at R=2.0, reaching 49.4% at R=3.0 and exceeding 93.4% when R≥10.0 (Fig. 10f). Notably, even small datasets can achieve near-perfect sensitivity provided they reach sufficient mutational depth (R>5.0, Figure S6). Moreover, our visualization of the informational landscape (Fig. 10) identifies distinct regimes of performance. We identify a robust sweet spot for discovery at sites with moderate to high substitution counts ($N_{subs}>10$) and low to moderate amino acid diversity ($N_{aa}\in [2,5]$). In this regime, the redundancy ratio exceeds 2.0, providing the mutational depth required to resolve specific property importance weights while minimizing the property-independent noise. Sensitivity in this region consistently exceeds 60%, even under strict FDR control (Fig. 10d).

Conversely, we detect three primary danger areas where biophysical signal is either extinguished or misidentified. The first is the invariance trap, located at the informational origin (Fig. 10c,d, bottom-left). Sites with few substitutions or extremely rigid constraints (λ>10) lack the requisite variation to characterize the biophysical filter, resulting in 0% power, highlighting that S-PRIME is fundamentally a comparative method. The second danger area is the toggling artifact observed at sites with high substitution counts but minimal amino acid diversity ($N_{aa}=2$; Fig. 10a). While repeated toggling between only two residues across deep phylogenies indicates constraint, attempting to map this restricted substitution pathway onto five independent properties creates an under-determined system. This complete separation causes the likelihood optimizer to diverge toward parameter boundaries, violating the asymptotic assumptions of Wilks’ Theorem and leading to inflated raw False Positive Rates (Fig. 10a). Formal logistic regression on 1,111,446 neutral simulation sites confirms that *R* is a strong predictor of spurious detection (AUC=0.76), with the predicted raw FPR rising from 1.8% at R=1.0 to 18.3% at R=5.0 and exceeding 65.0% when R≥20.0. Fortunately, the Benjamini-Hochberg correction effectively filters out these mathematically unidentifiable artifacts (Fig. 10b) because high-redundancy toggling sites are extremely rare under neutral evolution (<0.1% of simulated neutral sites have R>2.0). S-PRIME achieves peak parameter identifiability at sites exploring at least three distinct amino acid states. To address this, we implemented an automated warning in HyPhy v2.5.99 for sites where the redundancy ratio is low (R<2.0), flagging these positions for cautious interpretation. The third danger area is the saturation zone (Fig. 10c,d, top-right), where sites undergo high levels of turnover across a broad range of amino acids ($N_{aa}>10$), diluting the biophysical signal with property-independent noise and requiring substantial mutational depth to resolve (Fig. 10f).

Stratifying performance by individual datasets and selective regimes (online supplementary material, Figure S5) reveals a strong dependence of average detection power on informational content. We find that sensitivity is primarily a function of the total mutational depth (L×T); large, divergent alignments such as *rbcL* (RBCL), Mammalian Collagen (COLL), and HIV-1 RT (HVRT) achieve near-universal power across nearly all selective scenarios (Panel B). In contrast, sparsely sampled or low-divergence genes like Primate Lysozyme (LYSZ) and Mammalian AMELX (AMEL) remain largely underpowered even under intense selective regimes. This result confirms that the practical utility of S-PRIME is fundamentally constrained by the evolutionary information present in the multiple sequence alignment. A detailed breakdown of performance metrics and descriptive statistics (such as the redundancy ratio *R* and total substitutions) across all 24 benchmark datasets for a representative moderate-constraint scenario (easy_1) is provided in online supplementary material, Table S10. Importantly, the framework exhibits robust error control across this entire diversity of data regimes, and the empirical False Discovery Rate remains tightly calibrated to or below the nominal 0.10 threshold for virtually all benchmark genes (Fig. 10b), establishing S-PRIME as a reliable and statistically sound tool for biophysical characterization.

To evaluate sensitivity to model misspecification, we assessed S-PRIME on sequence data generated under an independent Mutation-Selection (MutSel) framework (online supplementary material, Tables S11 and S12). Under a gradient of selection intensity (Scenario S1), recovery power scaled directly with the strength of biophysical constraint, yielding a 13.8% overall recovery rate under Strong constraint ($N_{eff}=3.0$) in the 3-property model, which dropped to 4.9% and 2.2% under Medium ($N_{eff}=6.0$) and Weak ($N_{eff}=10.0$) regimes, respectively, due to low redundancy ratios (*R*). For sites subject to independent selection on three distinct properties (Scenario S2), S-PRIME demonstrated biophysical specificity, recovering Hydrophobicity (30.5%), Volume (36.9%), and Isoelectric Point (18.9%) constraints (online supplementary material, Table S11). Volume and Hydrophobicity cross-talk (e.g. 26.4% Hydrophobicity recovery in the Volume-selective partition) reflects the model’s projection of selection onto separate biophysical axes when property-specific turnover is limited, but the significance for the orthogonal Isoelectric Point property remained minimal (<5%). This cross-talk has a clear biophysical basis: purifying selection on a primary property frequently manifests as apparent diversifying selection (λ<0) for secondary properties. For example, in a Hydrophobicity-constrained partition, S-PRIME identified Hydrophobicity conservation ($\lambda_{\mathrm{Hydro}}=15.0$) coupled with significant Volume diversification ($\lambda_{\mathrm{Vol}}=-4.4$). Because the evolutionary process is restricted to hydrophobic residues (Val, Ile, Phe) which differ in size, the observed substitutions exhibit a higher variance in volume than expected under neutral models. S-PRIME thus characterizes the specific channels through which variation is permitted.

Evaluating compositional robustness (Scenarios S3–S5) using Frequency-Biased Neutral (FBN) controls—which share identical stationary distributions with their MutSel counterparts but lack selective dynamics (ω=1.0)—confirmed that S-PRIME is sensitive to rate dynamics rather than composition, with FBN false positive rates remaining ≤1.7% (online supplementary material, Table S11). S-PRIME was also robust to heterotachy (Scenario S6), yielding a 0.0% False Discovery Rate when purifying selection (ω=0.05) was restricted to a single terminal clade. For selection driven by an unmodeled property (Scenario S7, Aromaticity selection), S-PRIME detected significance in 50.5% of sites (primarily Volume conservation and Hydrophobicity diversification), reflecting the projection of aromatic side-chains (Phe, Tyr, Trp) onto the available property space.

Lastly, comparing the 3-property and 5-property models showed that the 5-property model (which incorporates nonrelevant secondary structure propensities) was more conservative under Holm-Bonferroni correction (M=5), yielding lower recovery rates for primary targets (e.g. S2 target recovery dropped from 30.5% to 10.8%; online supplementary material, Table S12). Nevertheless, it maintained high specificity, and sites under intense packing constraints were occasionally flagged for alpha-helix or beta-sheet propensities, capturing the geometric consequences of core burial.

## Discussion

In this study, we introduced the PRIME framework, a suite of codon-based evolutionary models that incorporate biophysical properties to characterize the mechanistic basis of natural selection. By applying these models across a taxonomically diverse benchmark and a genome-wide screen of nearly 19,000 mammalian genes, we identified the expected fundamental hierarchy of selective constraint: while core packing properties and beta-sheet scaffolds are rigidly conserved to maintain structural integrity, alpha-helix propensity and surface electrostatics serve as the primary substrates for evolutionary adaptation and episodic tuning. At the site-specific level, S-PRIME resolved the biophysical basis of key functional switches and identified constraints in residues that appear neutral by traditional dN/dS-based metrics. Furthermore, our validation against both experimental fitness landscapes and protein language models demonstrates that explicit physicochemical features effectively capture the biophysical principles of selection, providing an interpretable alternative to predictive frameworks that lack explicit mechanistic parameters.

The fundamental contribution of PRIME is its ability to resolve the specific biophysical constraints of natural selection, a goal that traces back to the 1970s (Grantham 1974) but has historically been overshadowed by the intuitive simplicity of biochemically agnostic dN/dS models (Yang 2006). By partitioning abstract substitution events into individual biophysical channels such as hydrophobicity, volume, and charge, we can define a more nuanced classification of molecular evolution. This resolution reveals that adaptation is often not a random exploration of sequence space, but rather a process of constrained diversification, where residues are driven to change in one property while being strictly prohibited from altering others. Conversely, PRIME identifies constraints at sites that appear evolutionarily neutral to rate-based models; at these positions, high turnover is permitted only among chemically similar residues, maintaining a functional constant despite sequence variation.

Our large-scale structural mapping provides a concrete physical manifestation of these dynamics, demonstrating that mammalian protein evolution is governed by a spatially resolved biophysical program where structural context dictates both the intensity and the chemical dimension of selective pressure. The structural core acts as a thermodynamic anchor, enforcing strict, multidimensional purifying selection on volume, charge, and hydrophobicity to preserve the integrity of the fold. Adaptability is systematically channeled to the solvent-exposed surface and flexible secondary structure motifs (such as alpha-helices), where the relaxation of packing constraints permits the fine-tuning of biophysical properties to mediate molecular interactions. Notably, when this spatial gradient is inverted or decoupled—as seen in the atypical surface hydrophobicity of *MRGPRX4* or the surface volume constraints of *GSTA3* observed during the genome-wide analysis—it highlights specialized functional interfaces and binding pockets rather than neutral drift. By resolving these spatial dynamics across the proteome, PRIME transforms abstract evolutionary rates into a physically grounded map of biophysical constraint and adaptation.

Since its initial implementation as a part of the HyPhy package over 10 years ago, the PRIME framework—specifically S-PRIME—has been applied across a diverse range of biological systems to resolve the specific physicochemical drivers of selection. These applications range from characterizing the adaptation of metabolic enzymes in extreme environments (Melo-Ferreira et al. 2014) and xenobiotic metabolism (Almeida et al. 2016) to dissecting the biophysical logic of viral drug resistance and immune escape (Grinev et al. 2016; Shepherd et al. 2017; Franzo et al. 2025). Furthermore, S-PRIME has identified critical constraints at protein-RNA interfaces (Mallik and Kundu 2017) and distinguished between adaptive and neutral drivers of molecular evolution in both innate immune receptors (Velová et al. 2018) and plant populations (Lee-Yaw et al. 2019). Collectively, these studies underscore the utility of transforming abstract evolutionary rates into concrete mechanistic hypotheses across diverse taxonomic scales and functional paradigms.

While standard rate-based tools (FEL, MEME, BUSTED) identify where and when selection acts, they are agnostic to its biophysical nature. PRIME partitions substitution events into biochemically interpretable rules, leading to three primary regimes of discrepancy. First, in unconstrained diversification (e.g. solvent-exposed loops), rate-based methods detect positive selection while PRIME yields null results because substitutions do not track specific properties. Second, PRIME identifies cryptic constraints at sites that appear neutral by rate metrics, where substitution turnover is permitted but limited to a chemically similar subset of residues. Finally, at diversifying sites, PRIME distinguishes between a chemical shift and a constrained exploration of a biophysical subspace. Integrating both frameworks allows researchers to separate unmodeled adaptive drift from targeted biophysical tuning.

This framework provides a connection between statistical phylogenetics and representation learning. While protein language models like ESM-2 (Lin et al. 2023) are robust predictors of fitness, the underlying drivers of their predictions remain difficult to interpret. The significant correlation between PRIME importance weights and the top principal components of PLM embeddings suggests that PRIME effectively characterizes the biophysical features learned by these models from substantial sequence databases. However, it is important to distinguish between interpretability and absolute predictive performance. While PLMs achieve higher zero-shot accuracy by capturing high-order sequence dependencies, PRIME provides a small-data alternative that resolves the mechanistic basis of selection from a single nucleotide sequence alignment. Moreover, by modeling physicochemical requirements rather than sequence frequency, PRIME reduces the impact of historical biases common to deep-learning approaches, identifying physicochemically viable substitutions that may be missing from training data but are permitted by the protein’s structural requirements.

Our simulation studies establish the statistical requirements for applying these biophysical models. The primary determinant of statistical power is the redundancy ratio ($R=N_{subs}/N_{aa}$), representing an approximation of how many times the evolutionary process has sampled the available state space. We find that R>2.0 serves as a critical threshold for meaningful biophysical inference. Researchers must also account for the loss of signal under purifying selection, an information-theoretic blind spot where constraints paradoxically become difficult to detect by extinguishing the very mutational signal required for characterization. Consequently, the greatest potential for discovery lies at sites with moderate to high turnover but restricted chemical breadth. Our framework exhibits tolerance to a degree of model misspecification, as evidenced by its ability to infer meaningful physicochemical correlates of substitution, even when the underlying selective landscape differs from modeling assumptions (e.g. Gaussian fitness under Mutation-Selection models), or when the primary driver (e.g. Aromaticity) is not explicitly modeled.

However, despite its gains in biophysical realism, PRIME operates under several simplifying assumptions that define the practical boundaries of its application:

1. **Mutational Depth:** The site-level optimization of 5–10 parameters requires substantial evolutionary turnover. While the omnibus test serves as a necessary safeguard against overfitting, we find that the test is generally conservative for the bulk of sites with low informational redundancy (Fig. 10C). Conversely, in regimes of extreme turnover with low diversity (toggling sites), the raw omnibus test can become anti-conservative, necessitating the methodical use of FDR correction (q≤0.10) which effectively eliminates most of these artifacts (Fig. 10B).
2. **Synonymous Rate Variation (SRV):** The current global (G-PRIME) and episodic (E-PRIME) implementations assume a constant synonymous rate (*α*) across the entire gene. For the genome-wide mammalian screen, we utilized G-PRIME with a global synonymous rate multiplier to maintain computational tractability, and direct comparabilty with traditional dN/dS methoods. While we acknowledge that unmodeled SRV can occasionally confound selection detection, our site-level S-PRIME implementation explicitly accounts for site-to-site variation, providing a more thorough validation for specific functional motifs where mutational hotspots may be a concern.
3. **Formulation of Positive Selection:** The functional form of the substitution rate (Equation (2)) is mathematically optimized for purifying selection (λ>0). In cases of diversifying selection (λ<0), the rate increases with physicochemical distance. While we impose a computational ceiling on these rates to prevent numerical divergence, this formulation implies that adaptation favors maximizing chemical difference. Biologically, however, positive selection often seeks a new structural optimum rather than infinite divergence. Consequently, negative *λ* weights should be interpreted as a continuum ranging from the active selection for physicochemical change to the total relaxation of structural constraints. In regions like IDRs, a signature of “diversification” may often reflect a permissive structural sieve that allows for neutral exploration of property space.
4. **Epistasis:** The current framework models each site as an independent entity. In reality, protein residues are highly cooperative, and the biophysical constraints at one site are contingent on the state of its neighbors. While S-PRIME effectively identifies the marginal selective pressures acting on individual residues, it does not explicitly model epistatic interactions. This limitation is shared by most site-specific frameworks, including Mutation-Selection (MutSel) models (Rodrigue et al. 2021), which characterize heterogeneous preferences across sites but typically assume site independence. Future work integrating PRIME’s biophysical grammar with hierarchical Bayesian models or explicit co-evolutionary frameworks could better resolve these structural dependencies and distinguish between alignment noise and genuine adaptation in rapidly evolving regions.
5. **Stationarity:** S-PRIME assumes a stationary process across the phylogeny. In contexts involving rapid selective sweeps or host-switching events, such as those observed in our H3N2 and HIV-RT analyses, this assumption represents a simplification of the underlying nonequilibrium dynamics, which may not fully capture transient shifts following recent selective sweeps or sudden environmental changes. While the framework could be extended to allow for site-specific branch-to-branch variation via random-effects mixture modeling, such an implementation would necessitate a substantial increase in mutational depth to remain statistically identifiable. We have therefore opted for a practical trade-off that prioritizes site-level biophysical resolution, while offering E-PRIME as a complementary tool for detecting episodic fluctuations at the gene-wide level. Given that stationary models remain the standard for molecular evolutionary analysis and have consistently delivered biologically meaningful results, we view the current implementation as a robust first-order approximation of the physicochemical rules governing sequence transition.
6. **Property Collinearity:** Fundamental biophysical attributes are often correlated. Our analysis reveals high collinearity between hydrophobicity, volume, and polarity. While the omnibus test remains a robust detector of selection, the precise partitioning of selective signal between these dimensions remains subject to uncertainty. Researchers should interpret individual property weights as part of a synergistic biophysical profile rather than isolated effects.

Based on our performance benchmarks, we suggest a hierarchical workflow tailored to the specific research objective. G-PRIME can be used to obtain a rapid, gene-wide biophysical fingerprint of a protein family, while E-PRIME is suited for investigating lineage-specific adaptation or episodic property shifts. For resolving the mechanistic basis of specific functional or drug-resistance motifs at individual sites, S-PRIME should be applied to positions with sufficient mutational depth, defined by a redundancy ratio R>2.0. For alignments with limited evolutionary information, simpler model configurations incorporating fewer properties are often worthwhile for resolving fundamental biophysical signals.

The ability to decode evolutionary history into concrete physical rules offers a powerful tool for prospective applications, such as identifying the biophysical constraints that channel viral immune escape or predicting the impact of mutations in uncharacterized proteins. By transforming the abstract study of evolutionary rates into a quantitative investigation of biophysical rules, PRIME provides a systematic framework for understanding the mechanistic drivers of genetic diversity.

## Supplementary Material

msag200_Supplementary_Data

## Data Availability

The PRIME framework is implemented as an open-source module in the standard library of HyPhy (v2.5.94+) and is freely available at https://github.com/veg/hyphy or installable via Conda (conda install -c conda-forge hyphy). All empirical benchmark datasets, simulated alignments, phylogenetic trees, processing scripts, and analysis workflows are publicly available in the project repository at https://github.com/veg/PRIME. Primary protein coordinates were retrieved from the RCSB Protein Data Bank (https://www.rcsb.org), mammalian gene alignments were obtained from the OrthoMaM database, and deep mutational scanning datasets were sourced from Lee et al. (2018).
