# Supplementary material for: The physicochemical basis of protein evolution: property-informed evolutionary models (PRIME)
Source: Mol Biol Evol. 2026 Aug 13;43(8):msag200. doi: 10.1093/molbev/msag200 (PMC13511143; doi:10.1093/molbev/msag200)
Supplement: Supplementary file 1 [file msag200_supplementary_data.pdf]

Supplementary Material for: The Physicochemical Basis of Protein Evolution: Property-Informed Evolutionary Models (PRIME)

Hannah Kim[1,2], Konrad Scheffler[3], Anton Nekrutenko[4], Darren P. Martin[5], Steven Weaver[1], Ben Murrell[6], and Sergei L. Kosakovsky Pond[1,2,*]

[1]Institute for Genomics and Evolutionary Medicine, Temple University, Philadelphia, PA, USA
[2]Department of Biology, Temple University, Philadelphia, PA, USA
[3]Current address: Illumina, Inc., San Diego, CA, USA
[4]Department of Biochemistry and Molecular Biology, The Pennsylvania State University, University Park, PA, USA
[5]Institute of Infectious Disease and Molecular Medicine, University of Cape Town, Cape Town, South Africa
[6]Department of Microbiology, Tumor and Cell Biology, Karolinska Institutet, Stockholm, Sweden
[*]To whom correspondence should be addressed. E-mail: spond@temple.edu

Supplementary Figures

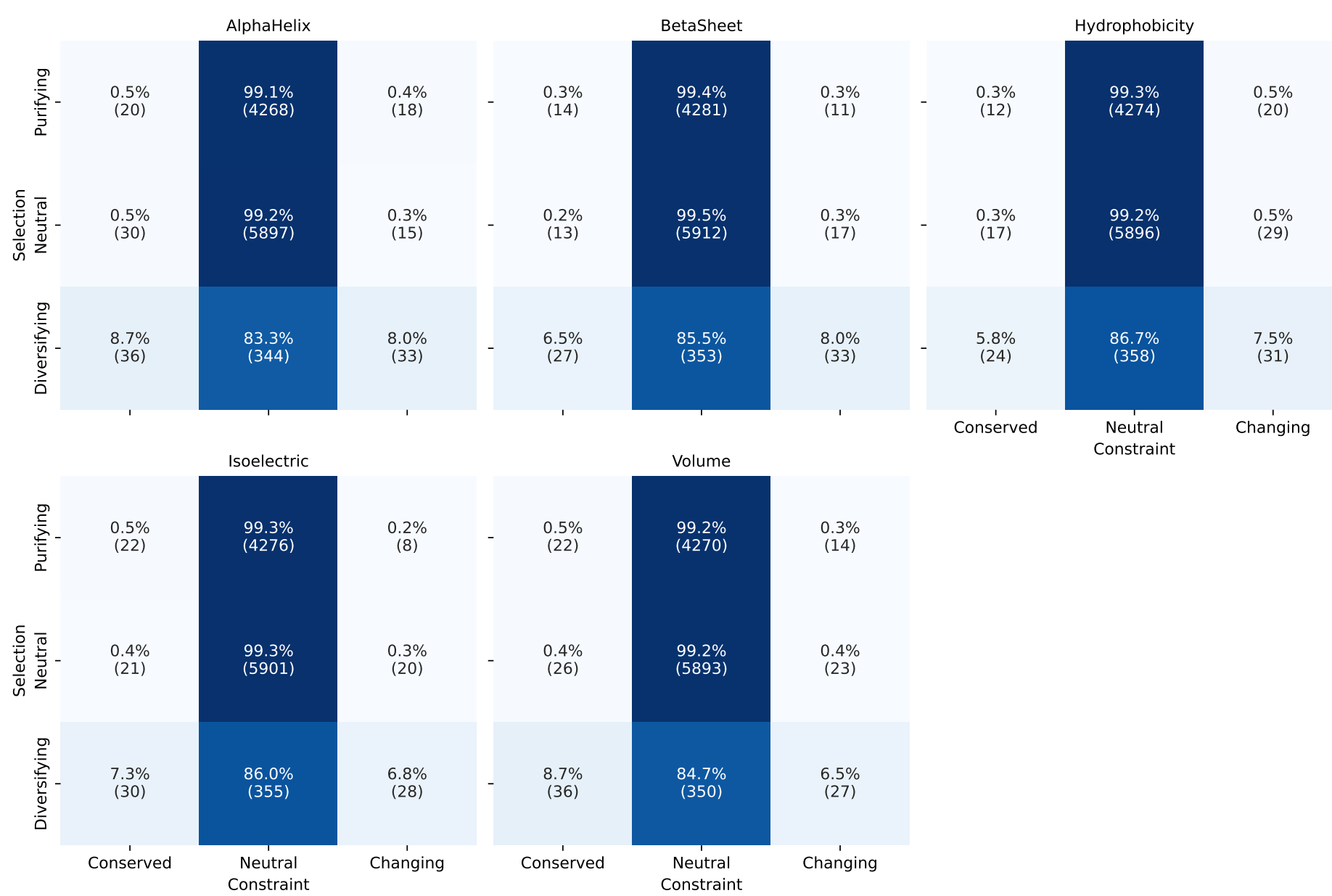

Figure S1: Conditional Probability of Physicochemical Constraints. Heatmap showing the percentage of sites within each selection class (Purifying, Neutral, Diversifying) that are Conserved, Neutral, or Changing for each physicochemical property. Raw site counts are shown in parentheses. Cryptic conservation is highlighted in the Neutral selection row, where 0.4% of sites exhibit significant property conservation despite lacking a rate-based signature of selection ($dN/dS \approx 1$). For sites subject to purifying selection or evolving neutrally, constraints represent either cryptic conservation ($\lambda > 0$) or tolerated variation ($\lambda < 0$). For sites subject to diversifying selection, the comparable representation of conserved ($\lambda > 0$) and changing ($\lambda < 0$) properties reflects channeled adaptation: positive selection for property change along one axis occurs against a background of purifying conservation on other axes (also see Supplementary Figure S2).

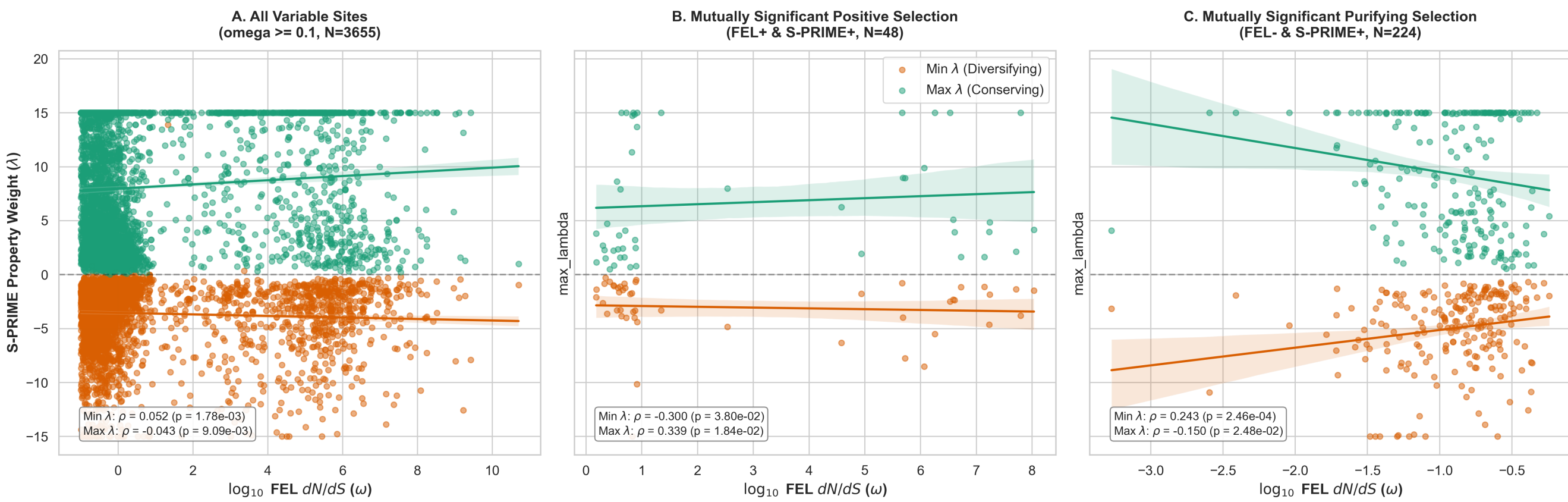

Figure S2: Relationship between Rate-Based Selection and S-PRIME Biophysical Constraints. (A) Correlation between FEL dN/dS (ω) and both $\min(\lambda)$ (orange) and $\max(\lambda)$ (green) across all variable sites ($\omega \geq 0.1$, $N = 3,655$), illustrating the global decoupling of rate acceleration and biophysical constraints. (B) Correlation for the subset of mutually significant positive selection sites (FEL $p \leq 0.05, \omega > 1$ and S-PRIME $q \leq 0.10$, $N = 48$), showing that higher positive selection rates correlate with both stronger diversifying selection ($\min(\lambda)$) and stronger purifying conservation ($\max(\lambda)$) on different properties. (C) Correlation for the subset of mutually significant purifying selection sites (FEL $p \leq 0.05, \omega < 1$ and S-PRIME $q \leq 0.10$, $N = 224$), demonstrating that as purifying selection relaxes (higher ω), property-specific constraints weaken (both $\min(\lambda)$ and $\max(\lambda)$ approach 0). Trend lines represent linear regressions on $\log_{10}(\omega)$, and Spearman rank correlations (ρ) with p-values are shown in the inset boxes.

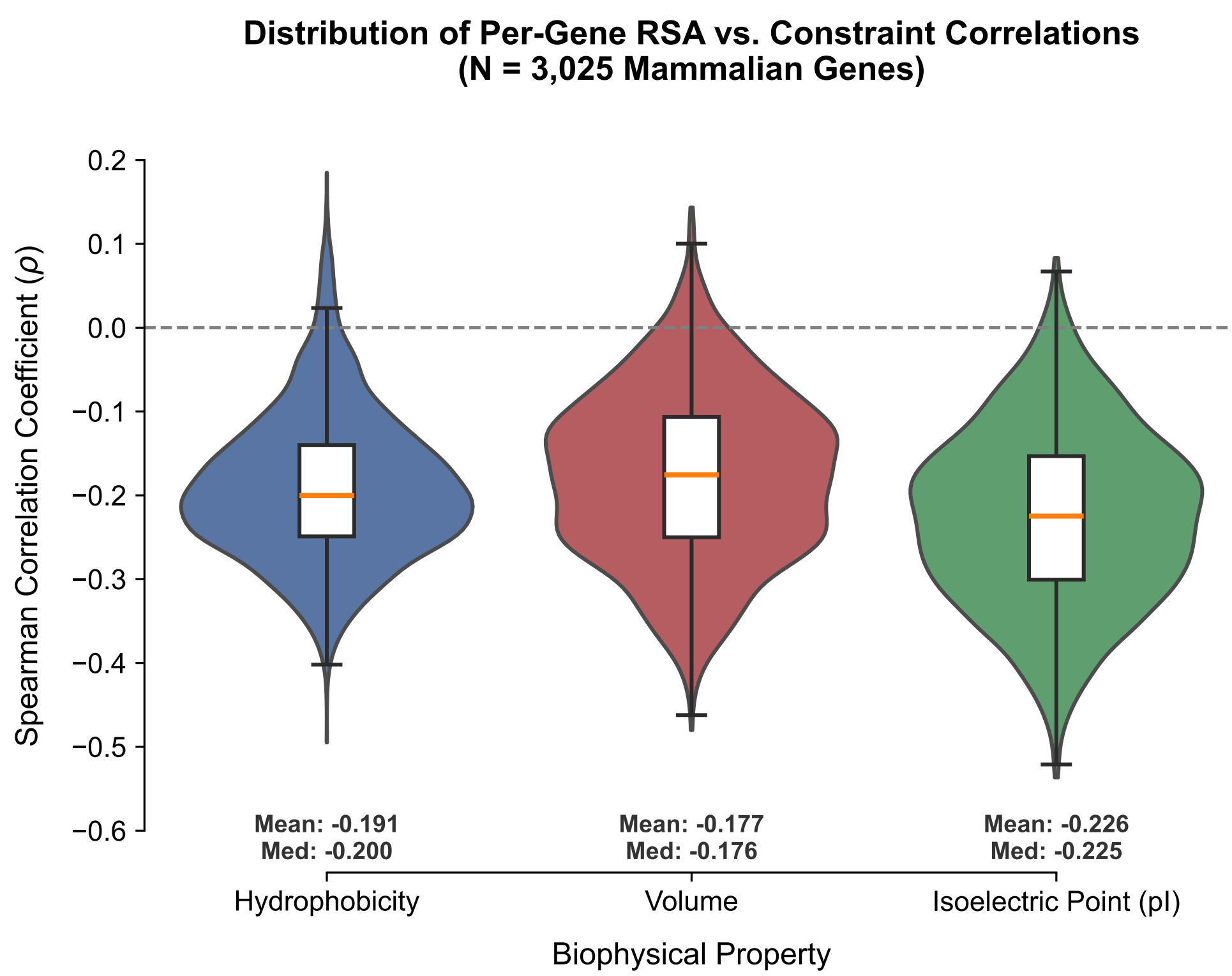

Figure S3: Genome-Wide Distribution of Per-Gene RSA vs. Constraint Correlations. Spearman rank correlation coefficients (ρ) between Relative Solvent Accessibility (RSA) and signed selection coefficients (λ) across all $3,025$ mammalian genes with at least 200 structurally mapped residues, plotted for Hydrophobicity (blue), Volume (red), and Isoelectric Point (green). The distributions are overwhelmingly negative (mean $\rho_{\text{Hydro}} = -0.191$, mean $\rho_{\text{Vol}} = -0.177$, and mean $\rho_{\text{pI}} = -0.226$), indicating that biophysical constraints universally decay from the core to the surface of proteins. Boxplots show the median (orange line), interquartile range (box), and $1.5 \times$ IQR (whiskers).

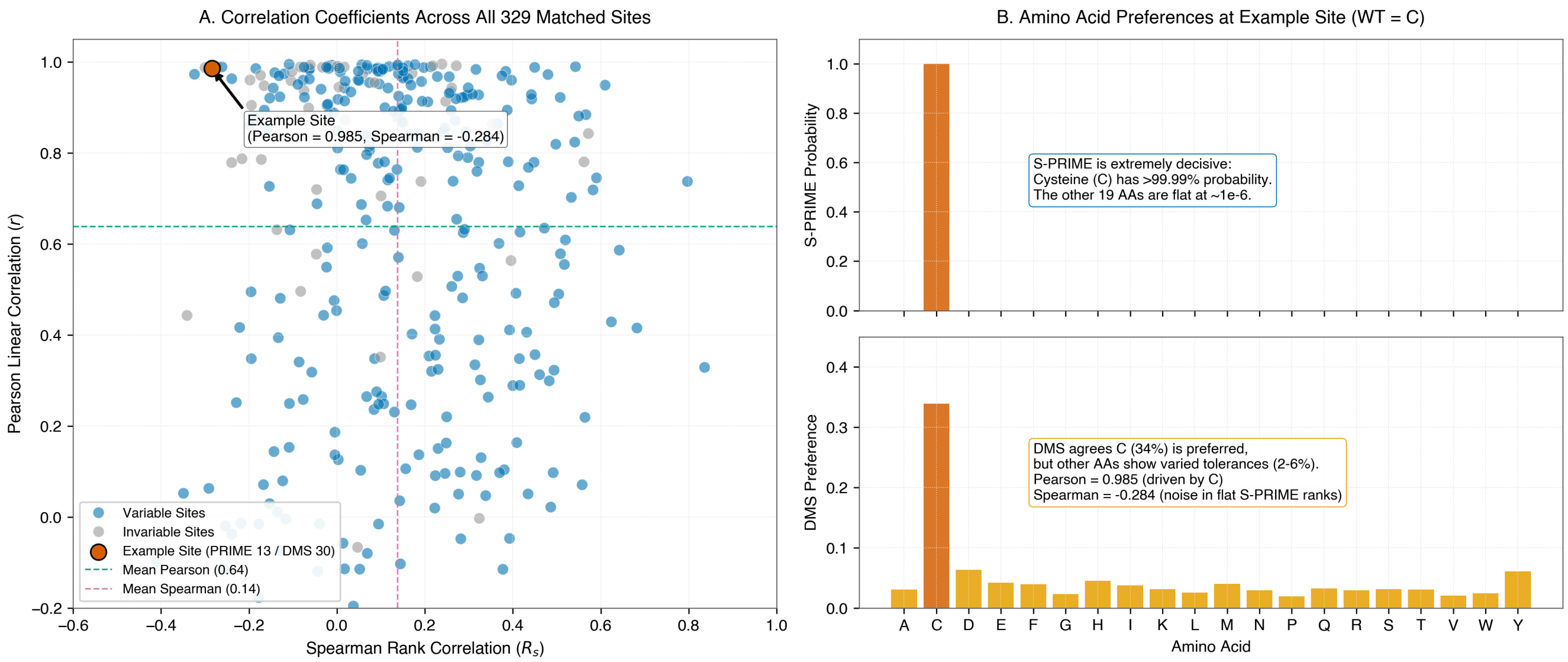

Figure S4: Conceptualizing Correlation Discrepancies in DMS Validation. (A) Distribution of site-specific Pearson linear correlation (r) and Spearman rank correlation (ρ) coefficients for H3N2 HA preferences. While Pearson values are high ($r \approx 0.61$), Spearman values are systematically lower ($\rho \approx 0.16$). (B) Comparison of S-PRIME predicted probabilities (top) and experimental DMS preferences (bottom) at an example site (WT=C). S-PRIME concentrates $> 90\%$ probability on the wild-type residue. Because the remaining 19 amino acids are flat at the model's floor (10^{-6}), their relative ranks are dominated by stochastic noise, which penalizes the rank-based Spearman correlation despite the high linear agreement on the primarily preferred residues.

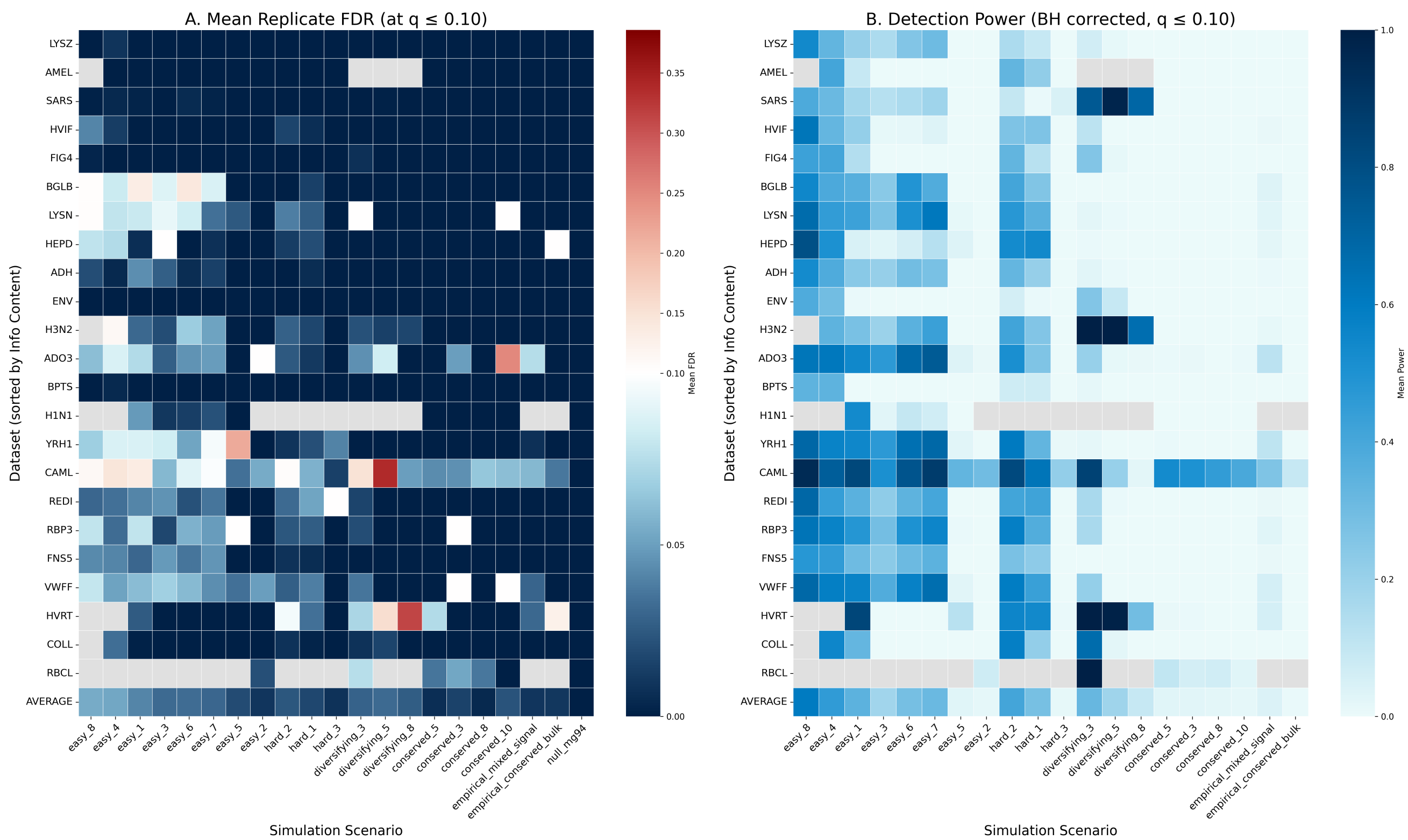

Figure S5: Dataset and scenario-specific performance S-PRIME performance on simulated data. (A) Empirical False Discovery Rate (FDR) calculated as the mean of replicate-level FDRs ($\frac{FP}{FP+TP}$) for each gene-scenario combination. The color scale is anchored at the nominal target $q \leq 0.10$ (Vik palette). (B) Mean detection power after Benjamini-Hochberg correction. The Y-axis is sorted by informational content ($L \times T$), ranging from minimal signal (LYSZ) to maximal signal (RBCL). Gray cells denote incomplete data (≤ 7 replicates). The final row (average) provides the scenario-wide mean across all benchmark genes.

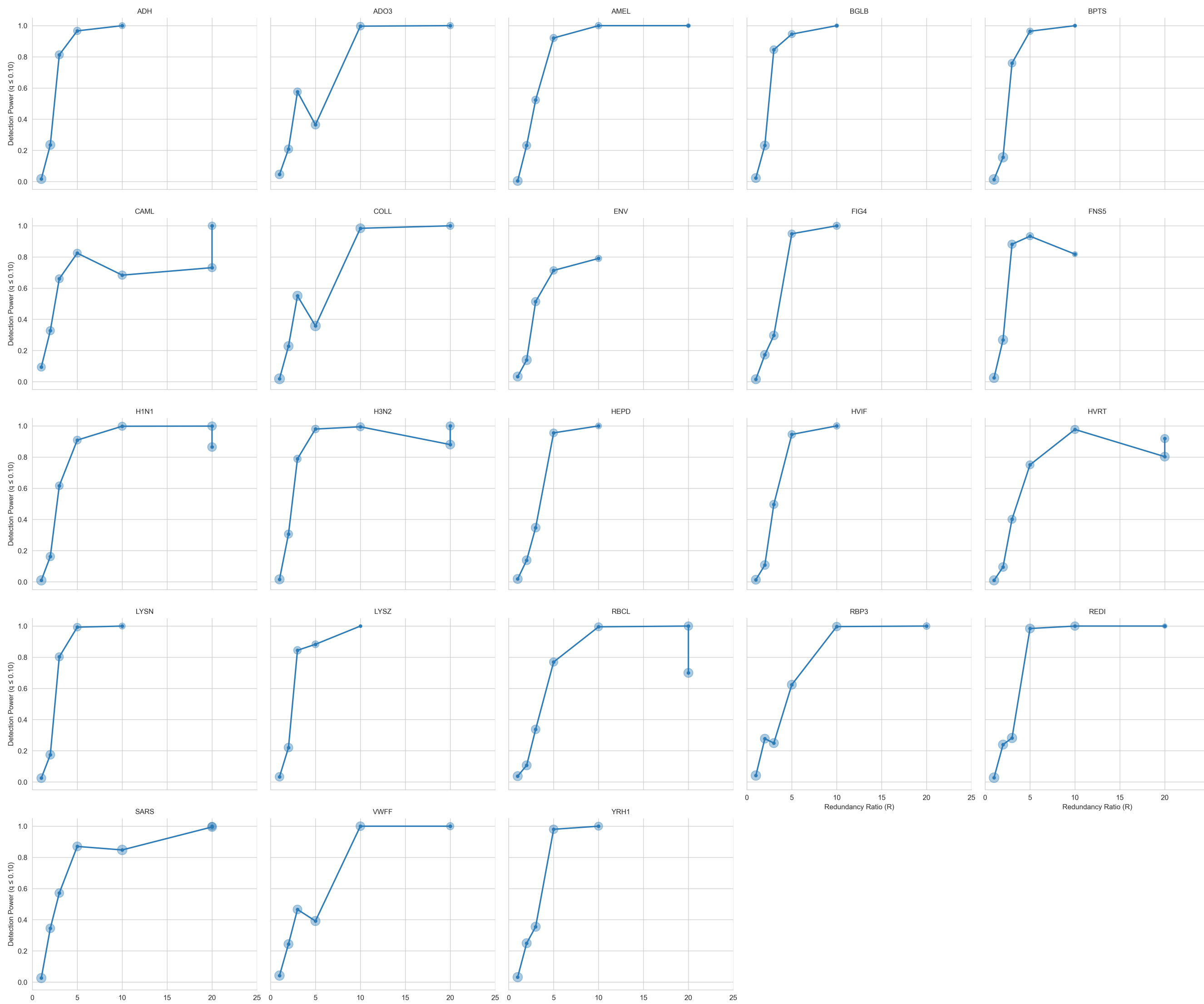

Figure S6: Detection power as a function of the redundancy ratio (R) for each of the 23 benchmark datasets. Detection is defined by the Benjamini-Hochberg corrected omnibus test ($q \leq 0.10$). Bubbles denote the relative number of simulation sites in each R bin. The consistent non-linear scaling across all genes confirms that informational depth is the primary driver of sensitivity, regardless of taxonomic origin or structural context.

Supplementary Tables

Table S1: Pairwise Associations and Mantel Test p-values between PRIME Biophysical Property Distances. For each pair of the five biophysical properties used in the 5-property PRIME model, we report the Pearson correlation coefficient (r) and the two-tailed empirical significance (p-value, in parentheses) calculated over the $\binom{20}{2} = 190$ pairwise distance vectors ($d_p(a, b) = |z_p(a) - z_p(b)|$). Statistical significance was assessed using the Mantel permutation test with 50,000 random node (amino acid) permutations to account for the structural dependency of the pairwise distances. Highly significant couplings ($p < 0.01$) are highlighted in bold.

Property	Hydrophobicity	Volume	Isoelectric Point	Alpha-Helix	Beta-Sheet
**Hydrophobicity**	1.0000	–	–	–	–
**Volume**	-0.1612 (0.0557)	1.0000	–	–	–
**Isoelectric Point**	0.1282 (0.2013)	-0.1372 (0.3570)	1.0000	–	–
**Alpha-Helix**	-0.0781 (0.3348)	0.1368 (0.1911)	-0.1089 (0.4341)	1.0000	–
**Beta-Sheet**	**0.3323 (0.0027)**	0.0880 (0.4060)	0.0451 (0.7494)	-0.0004 (0.9965)	1.0000

Table S2: Model fit comparison and dataset characteristics for 24 benchmark alignments. For each dataset, we report the number of sequences (N), the number of codons (L), the total tree length (T), and the PDB ID used for structural analysis. Fit improvement is shown as ΔLL relative to the $MG94$ baseline. Hierarchical property models are defined as follows: 2p (Hydrophobicity, Volume), 3p (+ Isoelectric Point), 4p (+ Alpha-Helix Propensity), and 5p (+ Beta-Sheet Propensity). The "Atchley" model utilizes the five orthogonal factors from Atchley et al. (2005). Models with improved fit according to AICc, BIC ($n = L$), BIC ($n = L \times N$), and head-to-head LRT ($p \leq 0.05$) are indicated by superscripts 1, 2, 3, and *, respectively. For datasets where all subscripts would be present we simply use "all."

Dataset	N	L	T	PDB	ΔLL(2p)	ΔLL(3p)	ΔLL(4p)	ΔLL(5p)	ΔLL Atchley	ΔLL CoRa
Primate Lysozyme	19	130	0.23	1LZ1 (130)	14.06^{all}	14.65^{all}	$15.39^{1,2,*}$	$17.02^{1,2,*}$	$9.30^{1,*}$	4.66^{all}
Mammalian AMELX	44	219	0.56	*None*	$7.38^{1,2,*}$	$13.63^{1,2,*}$	$14.24^{1,2,*}$	$14.25^{1,2,*}$	$9.78^{1,*}$	0.17
SARS-CoV-2 S	180	1284	0.12	6VXX (972)	2.30^{1}	$12.51^{1,2,*}$	$13.23^{1,*}$	$13.37^{1,*}$	$9.04^{1,*}$	1.88^{1}
HIV vif	29	192	0.91	4N9F (180)	10.38^{all}	$10.79^{1,2,*}$	$11.60^{1,2,*}$	23.12^{all}	$17.19^{1,2,*}$	0.27
SWS1 Opsin (Bats)	33	286	1.11	1U19 (280)	$3.90^{1,*}$	$4.24^{1,*}$	$6.90^{1,*}$	$8.05^{1,*}$	$9.17^{1,*}$	$2.61^{1,*}$
Mam. β-globin	17	144	2.25	2HHB (144)	55.34^{all}	79.07^{all}	97.06^{all}	112.68^{all}	80.83^{all}	13.84^{all}
Sperm lysin	25	134	2.54	2LIS (131)	28.18^{all}	29.39^{all}	32.67^{all}	33.95^{all}	25.16^{all}	6.77^{all}
Hepatitis D Ag	33	196	1.77	1A92 (49)	78.04^{all}	130.74^{all}	133.02^{all}	146.21^{all}	113.71^{all}	6.70^{all}
Drosophila adh	23	254	1.37	1A4U (254)	34.01^{all}	34.93^{all}	37.12^{all}	41.52^{all}	27.88^{all}	5.66^{all}
Encephalitis env	23	500	0.83	1SVB (389)	$4.83^{1,*}$	$6.65^{1,*}$	$10.08^{1,*}$	$10.18^{1,*}$	$9.65^{1,*}$	$2.86^{1,*}$
IAV H3N2 HA	349	329	1.41	4FNK (316)	$4.33^{1,*}$	$4.61^{1,*}$	$6.20^{1,*}$	$6.78^{1,*}$	$12.57^{1,*}$	0.01
ADORA3 (Mammals)	67	107	4.44	8X16 (95)	50.64^{all}	54.42^{all}	55.70^{all}	56.42^{all}	42.08^{all}	1.81^{1}
Streptococcus PTS	16	639	1.76	3QNQ (409)	72.49^{all}	73.12^{all}	73.15^{all}	73.16^{all}	41.67^{all}	9.36^{all}
IAV H1N1 HA	466	589	1.96	1RU7 (321)	75.17^{all}	76.10^{all}	76.14^{all}	79.53^{all}	37.58^{all}	32.38^{all}
Vertebrate Rhodopsin	38	330	3.76	1U19 (327)	166.67^{all}	274.05^{all}	275.37^{all}	283.73^{all}	184.25^{all}	$4.11^{1,2,*}$
Camelid VHH	212	96	14.13	1MEL (95)	50.01^{all}	66.05^{all}	66.05^{all}	73.13^{all}	98.47^{all}	18.42^{all}
Avian REDIC1	38	653	2.45	*None*	$7.18^{1,2,*}$	$10.07^{1,2,*}$	$10.63^{1,*}$	$13.97^{1,*}$	3.09	0.46
Mammalian RBP3	54	412	4.56	7JTI (158)	152.38^{all}	152.89^{all}	162.86^{all}	191.26^{all}	74.41^{all}	28.10^{all}
Flavivirus NS5	18	342	5.56	2J7U (328)	70.11^{all}	74.94^{all}	77.16^{all}	82.95^{all}	43.08^{all}	10.33^{all}
Mammalian VWF	62	392	5.16	1AUQ (376)	127.54^{all}	161.34^{all}	163.63^{all}	168.03^{all}	89.90^{all}	25.58^{all}
HIV rt	476	335	6.42	1RTD (327)	185.27^{all}	190.42^{all}	201.13^{all}	224.32^{all}	73.60^{all}	1.18^{1}
Mammalian COXI	21	510	5.08	5Z62 (506)	34.42^{all}	66.40^{all}	67.00^{all}	66.99^{all}	30.27^{all}	0.71
Mammalian Collagen	58	1459	2.08	5K31 (241)	353.54^{all}	495.37^{all}	498.48^{all}	501.42^{all}	310.10^{all}	43.60^{all}
rbcL (Plant RuBisCO)	483	466	10.98	1RXO (453)	445.33^{all}	667.72^{all}	667.72^{all}	669.91^{all}	295.42^{all}	43.31^{all}

Table S3: Selective Scenarios for S-PRIME Power Analysis. For each scenario, we report the biophysical importance factors (λ) applied to the 20% alternative partition. Properties: H: Hydrophobicity, V: Volume, P: Isoelectric Point (pI), α: Alpha-Helix, β: Beta-Sheet.

ID	Description	λ_H	λ_V	λ_P	λ_α	λ_β
easy_8	Easy: hydro++ vol+ pol– helix- sheet–	12.38	0.86	-5.02	-0.86	-2.37
easy_4	Easy: hydro– vol++ pol++ helix– sheet++	-4.91	3.85	1.16	-2.37	12.42
easy_1	Easy: hydro– vol++ pol- helix++ sheet–	-1.05	2.20	-0.66	11.37	-3.50
easy_3	Easy: hydro++ vol– pol++ helix++ sheet–	14.34	-1.64	9.52	2.18	-2.89
easy_6	Easy: hydro++ vol++ pol++ helix– sheet-	2.21	13.22	2.96	-4.16	-0.35
easy_7	Easy: hydro++ vol– pol++ helix- sheet++	4.94	-6.95	1.79	-0.60	13.80
easy_5	Easy: hydro– vol++ pol++ helix+ sheet-	-1.61	1.98	13.23	0.83	-0.87
easy_2	Easy: hydro+ pol+ helix- sheet+	0.95	0.09	0.63	-0.38	0.25
hard_2	Hard: hydro+ vol+ pol+ helix- sheet+	0.55	1.19	5.73	-2.56	1.07
hard_1	Hard: hydro+ vol+ pol- helix- sheet-	2.99	3.44	-1.69	-0.58	-0.99
hard_3	Hard: vol- pol+ helix+ sheet+	0.15	-0.55	0.54	1.00	0.40
diversifying_3	Diversifying: lambda=-3.0	0.00	-3.00	0.00	0.00	0.00
diversifying_5	Diversifying: lambda=-5.0	0.00	-5.00	0.00	0.00	0.00
diversifying_8	Diversifying: lambda=-8.0	0.00	-8.00	0.00	0.00	0.00
conserved_5	Conservation: lambda=5.0	0.00	5.00	0.00	0.00	0.00
conserved_3	Conservation: lambda=3.0	0.00	3.00	0.00	0.00	0.00
conserved_8	Conservation: lambda=8.0	0.00	8.00	0.00	0.00	0.00
conserved_10	Conservation: lambda=10.0	0.00	10.00	0.00	0.00	0.00
empirical_mixed_signal	Empirical Cluster 4: Strong Sheet conservation, moderate Vol diversification	-1.26	-1.99	3.41	0.27	9.39
empirical_conserved_bulk	Empirical Cluster 1: Moderate conservation on multiple properties	2.99	2.93	2.25	-0.14	-0.96
null_mg94	Strict Null: All lambdas = 0 (MG94)	0.00	0.00	0.00	0.00	0.00

Table S4: Functional and structural characteristics of the benchmark datasets.

Dataset	Function	Structural Features	Evolutionary Expectations
ADORA3 (Mammals) (Rodrigue et al., 2021)	G-protein coupled receptor	7-transmembrane alpha-helices	Conservation of core hydrophobicity for membrane insertion; variable loops
Avian REDIC1 (Jarvis et al., 2014)	NADH dehydrogenase subunit	Metabolic enzyme	Purifying selection on catalytic residues
Camelid VHH (Nguyen et al., 2002)	Single-domain antibody	Immunoglobulin fold (all-beta sandwich)	Conservation of beta-sheet propensity; diversifying selection on loops (CDRs)
Drosophila adh (Yang et al., 2000b)	Alcohol dehydrogenase	Alpha/Beta Rossmann fold	Conservation of catalytic triad and nucleotide binding domain
Encephalitis env (Yang et al., 2000b)	Viral envelope protein	Class II fusion protein (beta-sheet rich)	Surface exposure drives immune evasion; membrane fusion imposes structural constraints
Flavivirus NS5 (Kuno et al., 1998)	RNA-dependent RNA polymerase	Finger-palm-thumb domain architecture	Strict conservation of catalytic core
Hepatitis D Ag (Anisimova and Yang, 2004)	Viral capsid / RNA binding	Coiled-coil dimerization domain	Positive charge required for RNA interaction
HIV rt (Seoighe et al., 2007)	Reverse transcriptase	Heterodimer; mixed alpha/beta	Drug resistance mutations; conservation of polymerase active site
HIV vif (Yang et al., 2000b)	Viral infectivity factor	Ubiquitin ligase recruitment	Protein-protein interaction interfaces
IAV H1N1 HA (Tamuri and Dos Reis, 2022)	Viral hemagglutinin	Class I fusion (alpha-helical stem)	Stem region highly conserved (helix); head region variable (immune escape)
IAV H3N2 HA (Bush et al., 1999)	Viral hemagglutinin	Class I fusion (alpha-helical stem)	Stem region highly conserved (helix); head region variable (immune escape)
Mam. β-globin (Yang et al., 2000b)	Oxygen transport	Globin fold (all-alpha)	Strict requirement for alpha-helix propensity; heme binding pocket
Mammalian AMELX (Randall et al., 2022)	Tooth enamel formation	Intrinsically disordered protein (IDP)	Conservation of properties mediating biomineralization and nanosphere assembly
Mammalian Collagen (Mu et al., 2021)	Structural protein (Type I)	Triple helix (Gly-X-Y repeats)	Extreme conservation of volume (Gly requirement) and secondary structure propensity
Mammalian COXI	Cytochrome c oxidase subunit I	12-transmembrane alpha-helices	Conservation of hydrophobicity in TM domains; mitochondrial membrane insertion
Mammalian RBP3 (Rodrigue et al., 2021)	Retinol binding protein	Lipocalin fold (beta-barrel)	Hydrophobic pocket for retinol binding; conservation of beta-strands
Mammalian VWF (Rodrigue et al., 2021)	Blood coagulation factor	Multidomain glycoprotein	Shear-stress sensing; interaction with collagen and platelets
Primate Lysozyme (Yang, 1998)	Antibacterial enzyme	Alpha+Beta fold	Adaptive evolution in foregut fermenters (stability at low pH)
rbcL (Plant RuBisCO) (Tamuri and Dos Reis, 2022)	Carbon fixation	Alpha/Beta barrel (TIM barrel)	Extreme conservation of catalytic core; thermal stability constraints
SARS-CoV-2 S (Martin et al., 2021)	Viral spike glycoprotein	Class I fusion (alpha-helical coiled-coil)	Receptor binding domain (RBD) evolution; fusion machinery conservation
Sperm lysin (Yang et al., 2000a)	Gamete recognition/lysis	Four-helix bundle	Rapid evolution due to sexual conflict; conservation of helical structure
Streptococcus PTS (Dunn et al., 2019)	Sugar transporter	Membrane permease	Transmembrane helices
SWS1 Opsin (Bats) (Wertheim et al., 2015)	UV-sensitive opsin	7-transmembrane alpha-helices	Spectral tuning via amino acid shifts
Vertebrate Rhodopsin (Yokoyama et al., 2008)	Dim-light vision GPCR	7-transmembrane alpha-helices	Spectral tuning via electrostatic environment (charge)

Table S5: Estimated property importance weights (λ) for the 5-property PRIME model across 24 benchmark alignments. Datasets are sorted by the product of sequence length and tree length ($L \times T$). λs statistically significantly different from zero (Likelihood Ratio Test) are bolded and colored (Blue for conserved, Red for diversifying). Significance levels: * $p < 0.05$, ** $p < 0.01$, *** $p < 0.001$.

Dataset	Hydrophobicity	Volume	Isoelectric Point	Alpha-Helix	Beta-Sheet
Primate Lysozyme	0.55	0.94**	-0.22	0.19	0.59
Mammalian AMELX	0.13***	0.35***	0.37***	0.12	0.01***
SARS-CoV-2 S	0.19*	-0.05	-0.23***	0.09	0.04
HIV vif	0.07	0.25**	-0.04	0.02	0.51
SWS1 Opsin (Bats)	0.04	0.12	-0.02	0.15	0.15
Mam. β-globin	0.73***	0.53***	0.45***	-0.48***	0.66
Sperm lysin	0.21**	0.39***	0.07	-0.17*	0.13
Hepatitis D Ag	0.35***	0.43***	0.45***	-0.17**	0.50
Drosophila adh	0.56***	0.21	0.07	0.21*	0.38
Encephalitis env	0.57**	0.15	0.20*	-0.32*	-0.07
IAV H3N2 HA	0.03	0.11*	-0.03	0.08	0.07
ADORA3 (Mammals)	0.51***	0.26***	0.12*	0.09	0.10
Streptococcus PTS	0.43***	0.72***	-0.06	-0.02	-0.01
IAV H1N1 HA	0.16***	0.34***	-0.03	-0.01	0.12
Vertebrate Rhodopsin	0.68***	0.42***	0.85***	-0.08	0.26
Camelid VHH	0.16***	0.13***	0.14***	0.00	0.14
Avian REDIC1	-0.12***	0.08*	0.05*	-0.04	0.09
Mammalian RBP3	0.59***	0.20***	-0.02	-0.20***	0.32
Flavivirus NS5	0.22**	0.72***	0.16**	-0.16*	0.28
Mammalian VWF	0.43***	0.27***	0.19***	-0.09**	0.11
HIV rt	0.20***	0.72***	0.06**	-0.21***	0.34
Mammalian COXI	0.10	0.83***	0.74***	0.09	0.01
Mammalian Collagen	-0.01	1.20***	0.53***	-0.09**	-0.10*
rbcL (Plant RuBisCO)	0.02	0.58***	0.35***	0.01	0.05

Table S6: 5-property E-PRIME Parameter Estimates. For each dataset, we report the estimated property importance weights (λ) for the two mixture components. The weight of each component is shown in the second column. Values significantly different from zero (LRT $p < 0.05$) are shown in bold. Values at the upper bound of 10.00 are indicated with a dagger symbol.

Dataset	Weight	Hydrophobicity	Volume	Isoelectric Pt	Alpha Helix	Beta Sheet
Primate Lysozyme	0.80	-0.19	-3.05	**-2.85**	7.61	10.00†
	0.20	**0.95**	1.38	0.07	-0.01	0.23
Mammalian AMELX	0.97	**0.38**	0.36	**1.78**	-0.12	-0.22
	0.03	**0.09**	0.78	**-0.38**	0.55	0.61
SARS-CoV-2 S	0.97	10.00†	-5.54	-3.60	2.78	7.65
	0.03	0.10	0.00	-0.15	0.12	0.02
HIV vif	0.93	**0.95**	0.59	**0.20**	-0.22	0.92
	0.07	-0.36	-0.07	**-0.20**	0.09	0.18
SWS1 Opsin (Bats)	0.79	2.18	8.31	-0.18	10.00†	1.47
	0.21	-0.03	0.04	-0.03	-0.06	0.05
Mam. β-globin	0.91	**1.36**	0.95	**0.18**	0.14	0.22
	0.09	**0.67**	0.43	**1.26**	-0.88	1.88
Sperm lysin	0.80	9.31	-4.29	10.00†	10.00†	9.70
	0.20	0.34	0.56	**0.11**	-0.56	0.25
Hepatitis D Ag	0.90	3.48	4.25	2.83	-1.92	1.02
	0.10	0.31	0.21	**0.55**	0.22	0.50
Drosophila adh	0.94	**10.00†**	1.69	**10.00†**	-0.10	0.76
	0.06	0.62	0.34	0.05	0.21	0.64
Encephalitis env	0.62	**5.11**	10.00†	5.58	-1.15	2.02
	0.38	0.61	-0.02	**0.15**	-0.30	-0.13
IAV H3N2 HA	0.96	**0.72**	0.32	**-0.73**	-0.94	2.06
	0.04	**-0.31**	0.26	**0.42**	0.56	-0.26
ADORA3 (Mammals)	0.71	9.00	0.74	-0.04	-3.48	6.88
	0.29	0.35	0.40	0.11	0.42	-0.12
Streptococcus PTS	0.87	10.00†	0.40	0.61	-0.20	-0.41
	0.13	0.60	1.37	**-0.12**	-0.14	0.11
IAV H1N1 HA	0.87	**-0.78**	1.36	**7.74**	2.03	-1.24
	0.13	**0.12**	0.38	**-0.10**	-0.09	0.21
Vertebrate Rhodopsin	0.96	1.55	2.06	**8.63**	-0.21	1.18
	0.04	**1.19**	0.48	0.83	-0.52	0.05
Camelid VHH	0.95	**0.35**	0.24	0.13	-0.08	0.11
	0.05	-0.16	-0.06	**0.22**	0.05	0.42
Avian REDIC1	0.52	**-0.12**	0.07	**0.07**	-0.06	0.08
	0.48	**8.85**	10.00†	**-5.10**	6.51	7.17
Mammalian RBP3	0.82	2.04	0.64	**0.83**	-1.56	-0.75
	0.18	**0.37**	0.03	-0.28	0.57	0.89
Flavivirus NS5	0.90	-0.52	8.18	10.00†	10.00†	7.22
	0.10	0.49	1.62	0.25	-0.77	0.30
Mammalian VWF	0.73	8.89	1.50	**10.00†**	0.29	-0.60
	0.27	**0.40**	0.33	**0.16**	-0.15	0.12
HIV rt	0.96	**10.00†**	-0.54	**-0.55**	-4.84	2.34
	0.04	-0.03	1.12	**0.17**	0.00	0.21
Mammalian COXI	0.96	**-0.77**	1.99	**5.15**	10.00†	-2.65
	0.04	**0.40**	1.43	**0.84**	-0.31	-0.32
Mammalian Collagen	0.95	**-1.88**	10.00†	**4.37**	-2.76	-0.60
	0.05	**0.43**	0.76	**0.48**	0.14	-0.42
rbcL (Plant RuBisCO)	0.97	-1.74	7.90	**10.00†**	-0.70	-0.33
	0.03	**0.28**	0.29	**0.31**	0.03	-0.04

Table S7: Site Classification Counts across Benchmark Datasets. Sites were classified based on the decision tree in Figure **??**, integrating MEME selection detection ($p \leq 0.05$) with PRIME physicochemical structure (FDR $\leq$ 0.10). *Adaptive* classes (left) correspond to sites under diversifying selection; *Neutral/Purifying* classes (right) correspond to sites without rate elevation. Abbreviations: Constr. (Constrained), Div. (Diversification), Unconstr. (Unconstrained), Drv. (Drivers), Neut. (Neutral).

	**Adaptive (MEME +)**				**Neutral / Purifying (MEME -)**			
**Dataset**	**Constr. Div.**	**Driven Div.**	**Complex Div.**	**Unconstr. Div.**	**Cryptic Constr.**	**Cryptic Drv.**	**Complex Neut.**	**Non-Sig.**
rbcL (Plant RuBisCO)	47	10	15	11	43	11	41	288
Mammalian Collagen	9	3	7	30	15	5	15	1375
Mammalian VWF	4	0	2	1	30	4	28	323
HIV rt	12	3	4	3	14	7	19	273
Vertebrate Rhodopsin	1	2	0	9	15	8	24	271
Camelid VHH	8	4	8	9	14	2	11	40
Avian REDIC1	0	0	1	45	2	1	1	603
Sperm lysin	10	0	3	13	9	3	7	89
Hepatitis D Ag	3	0	4	12	5	1	5	166
IAV H1N1 HA	4	1	2	17	1	1	1	562
IAV H3N2 HA	2	0	3	12	2	1	4	305
Mammalian RBP3	0	0	1	3	2	3	8	395
Flavivirus NS5	1	0	0	1	7	1	3	329
HIV vif	0	0	0	8	1	1	3	179
ADORA3 (Mammals)	0	0	0	1	4	2	3	97
Mam. beta-globin	0	0	0	0	8	1	1	134
SARS-CoV-2 S	0	0	0	7	2	0	0	1275
Streptococcus PTS	0	0	0	9	0	0	0	630
Drosophila adh	0	0	0	8	0	0	0	246
Mammalian COXI	0	0	0	2	0	1	0	507
SWS1 Opsin (Bats)	0	0	0	3	0	0	0	283
Encephalitis env	0	0	0	1	1	0	0	498
Mammalian AMELX	0	0	0	2	0	0	0	217
Primate Lysozyme	0	0	0	0	0	0	0	130

Table S8: PRIME vs. DMS Validation for Significant H3N2 Sites. Properties shown are individually significant after the full two-stage hierarchical correction ($q < 0.05$, controlling for selective inference across the entire gene). 'DMS Residues' account for 80 percent of the preference mass. A negative Z-score confirms clustering in property space (Constraint). Consistent with Figure 7, sites located in the buried core (e.g., Site 226) exhibit higher importance weights and more restricted experimental variance.

Site	Property	λ	Type	Aln	DMS (WT **bold**)	N_{eff}	Var	Z
186	Polarity (pI)	13.29	Conserved	CGISV	ASN**G**CEMVP	11.3	1.28	-0.95
226	Hydrophobicity	-2.49	Driven	ILQV	TSQVMH**I**FN	13.3	8.46	0.21
	Volume	-1.81	Driven				1026.80	-1.22
	Polarity (pI)	15.00	Conserved				0.91	-1.59
	Helix Propensity	11.82	Conserved				0.05	-1.14

Table S9: S-PRIME Simulation Results by selective scenario. We report the mean False Positive Rate (FPR) and Power (sensitivity) averaged over all replicates (N) for both raw ($\alpha = 0.05$) and Benjamini-Hochberg corrected ($q \leq 0.10$) omnibus tests. For the `null_mg94` scenario, where all sites are neutral, results from both partitions are pooled into a single FPR estimate.

Scenario	N	Raw FPR	Raw Power	BH FPR	BH Power
easy_8	195	0.035	0.646	0.011	0.603
easy_4	210	0.033	0.493	0.008	0.456
easy_1	220	0.027	0.451	0.006	0.349
easy_3	221	0.023	0.216	0.003	0.183
easy_6	221	0.027	0.328	0.005	0.289
easy_7	219	0.024	0.354	0.005	0.315
easy_5	223	0.022	0.093	0.000	0.031
easy_2	222	0.015	0.064	0.000	0.017
hard_2	216	0.018	0.480	0.004	0.408
hard_1	216	0.017	0.362	0.002	0.284
hard_3	220	0.014	0.057	0.000	0.014
diversifying_3	217	0.023	0.508	0.006	0.322
diversifying_5	213	0.026	0.308	0.006	0.187
diversifying_8	211	0.029	0.194	0.005	0.091
conserved_5	226	0.015	0.082	0.000	0.029
conserved_3	226	0.016	0.082	0.000	0.025
conserved_8	226	0.015	0.064	0.000	0.023
conserved_10	226	0.015	0.055	0.001	0.019
empirical_mixed_signal	221	0.014	0.075	0.000	0.039
empirical_conserved_bulk	219	0.016	0.023	0.000	0.005
null_mg94	228	0.019	—	0.000	—

Table S10: Detailed S-PRIME Performance and Descriptive Statistics by Dataset for Scenario `easy_1` ($\lambda \approx 11$). $R = N_{subs}/N_{aa}$ denotes the Redundancy Ratio. FPR and Power are reported for raw ($\alpha = 0.05$) and BH-corrected ($q \leq 0.10$) tests.

Dataset	N	FPR (Raw)	FPR (BH)	Power (Raw)	Power (BH)	Med R_{null}	Med R_{alt}	Med S_{null}	Med S_{alt}
AMELX	10	0.002	0.000	0.286	0.091	0.5	1.4	1	8
Bacterial_PTS_trehalose_transporter_suIII	10	0.002	0.000	0.107	0.002	0.5	0.8	1	4
COL1A1	10	0.005	0.000	0.478	0.329	0.5	1.7	1	9
ENCenv	10	0.000	0.000	0.126	0.005	0.0	1.0	0	3
Fig4E	10	0.001	0.000	0.302	0.138	0.5	1.3	1	6
HIV_RT	10	0.022	0.006	0.885	0.834	1.3	5.1	6	48
HIVvif	10	0.007	0.000	0.395	0.213	0.7	1.5	2	8
HepatitisD	10	0.018	0.001	0.287	0.048	0.8	1.9	3	19
IAV-human-H1N1-HA	8	0.031	0.006	0.604	0.538	1.0	2.5	3	16
InfluenzaA	8	0.032	0.003	0.335	0.280	1.0	0.5	3	1
REDIC1	10	0.029	0.004	0.438	0.352	1.0	1.0	4	2
SARS-CoV-2-spike	9	0.003	0.000	0.177	0.172	0.0	0.0	0	0
adh	10	0.037	0.003	0.325	0.239	0.8	0.5	3	1
adora3	10	0.046	0.012	0.605	0.545	1.7	1.3	11	4
bglobin	10	0.070	0.015	0.514	0.362	1.1	1.2	7	3
camelid	10	0.072	0.037	0.830	0.825	3.7	6.6	41	26
flavNS5	10	0.036	0.003	0.428	0.307	1.0	1.0	4	2
lysin	10	0.043	0.009	0.559	0.430	1.2	1.2	7	4
lysozyme	10	0.006	0.000	0.265	0.208	0.5	0.5	1	1
rbcL	5	0.050	0.015	0.726	0.698	3.0	3.1	24	12
rbp3	10	0.040	0.010	0.559	0.482	1.4	1.3	8	4
vwf	10	0.042	0.010	0.618	0.567	1.6	1.5	10	5
yokoyama.rh1.cds.mod.1-990	10	0.046	0.013	0.639	0.548	1.4	1.5	10	5

Table S11: Multi-Scenario Property Recovery (MSPR) for the 3-Property model. Overall significance is assessed using Omnibus LRT ($q \leq 0.1$), and individual properties are tested using hierarchical Holm-Bonferroni correction ($p \leq \alpha R/N$).

Scenario	Partition	Description	Overall	Hydrophobicity	Volume	Isoelectric
S1 Gradient	Strong	$N_{eff} = 3.0$	13.8%	1.8%	1.9%	0.0%
S1 Gradient	Med	$N_{eff} = 6.0$	4.9%	1.5%	1.0%	0.2%
S1 Gradient	Weak	$N_{eff} = 10.0$	2.2%	0.6%	0.4%	0.8%
S1 Gradient	Neutral	Neutral Control	3.7%	2.2%	0.6%	1.1%
S2 Orthogonal	Hydro	Hydrophobicity Target	51.1%	30.5%	26.4%	0.9%
S2 Orthogonal	Vol	Volume Target	42.2%	18.9%	36.9%	4.9%
S2 Orthogonal	pI	pI Target	62.4%	6.1%	33.6%	18.9%
S2 Orthogonal	Neutral	Neutral Control	3.7%	1.1%	1.0%	1.4%
S3 CompHydro	MutSel	Hydrophobicity Target	15.7%	3.9%	3.4%	0.2%
S3 CompHydro	FBN	Frequency-Biased Neutral	0.8%	0.2%	0.1%	0.3%
S3 CompHydro	Neutral	Neutral Control	3.2%	2.0%	0.4%	0.9%
S4 CompVol	MutSel	Volume Target	9.9%	3.0%	7.9%	0.4%
S4 CompVol	FBN	Frequency-Biased Neutral	0.5%	0.1%	0.1%	0.3%
S4 CompVol	Neutral	Neutral Control	1.1%	0.4%	0.7%	0.3%
S5 CompPI	MutSel	pI Target	44.2%	2.3%	12.4%	8.0%
S5 CompPI	FBN	Frequency-Biased Neutral	1.7%	0.5%	0.5%	0.5%
S5 CompPI	Neutral	Neutral Control	3.2%	0.9%	0.8%	0.8%
S6 Heterotachy	Het	Terminal Clade Purifying	0.0%	0.0%	0.0%	0.0%
S6 Heterotachy	Neutral	Neutral Control	0.0%	0.0%	0.0%	0.0%
S8 Aromatic	Aromatic	F, Y, W Target (Unmodeled)	33.7%	9.9%	3.7%	1.5%
S8 Aromatic	NonArom	Non-aromatic Target	4.1%	1.0%	1.9%	1.3%
S8 Aromatic	Neutral	Neutral Control	1.3%	0.3%	0.4%	0.6%

Table S12: Multi-Scenario Property Recovery (MSPR) for the 5-Property model. Overall significance is assessed using Omnibus LRT ($q \leq 0.1$), and individual properties are tested using hierarchical Holm-Bonferroni correction ($p \leq \alpha R/N$).

Scenario	Partition	Description	Overall	Hydrophobicity	Volume	Isoelectric	Alpha Helix	Beta Sheet
S1 Gradient	Strong	$N_{eff} = 3.0$	7.9%	0.1%	0.1%	0.0%	0.0%	0.0%
S1 Gradient	Med	$N_{eff} = 6.0$	4.1%	0.2%	0.2%	0.1%	0.1%	0.3%
S1 Gradient	Weak	$N_{eff} = 10.0$	2.7%	0.3%	0.4%	0.6%	0.2%	0.3%
S1 Gradient	Neutral	Neutral Control	5.3%	0.9%	0.2%	0.5%	0.5%	0.4%
S2 Orthogonal	Hydro	Hydrophobicity Target	60.0%	10.8%	9.5%	1.8%	2.4%	4.1%
S2 Orthogonal	Vol	Volume Target	41.5%	2.1%	11.3%	1.6%	2.3%	2.6%
S2 Orthogonal	pI	pI Target	62.6%	2.0%	2.1%	6.3%	2.2%	2.0%
S2 Orthogonal	Neutral	Neutral Control	7.2%	0.9%	0.8%	1.0%	1.0%	1.1%
S3 CompHydro	MutSel	Hydrophobicity Target	9.5%	0.5%	0.5%	0.2%	0.2%	0.2%
S3 CompHydro	FBN	Frequency-Biased Neutral	1.9%	0.3%	0.2%	0.3%	0.3%	0.3%
S3 CompHydro	Neutral	Neutral Control	4.6%	0.6%	0.2%	0.4%	0.5%	0.3%
S4 CompVol	MutSel	Volume Target	10.1%	0.6%	1.8%	0.3%	0.5%	0.5%
S4 CompVol	FBN	Frequency-Biased Neutral	1.1%	0.2%	0.2%	0.2%	0.1%	0.2%
S4 CompVol	Neutral	Neutral Control	2.2%	0.3%	0.3%	0.1%	0.2%	0.1%
S5 CompPI	MutSel	pI Target	33.3%	1.0%	0.8%	2.5%	0.6%	1.0%
S5 CompPI	FBN	Frequency-Biased Neutral	2.8%	0.5%	0.6%	0.6%	0.5%	0.6%
S5 CompPI	Neutral	Neutral Control	4.8%	0.6%	0.5%	0.5%	0.6%	0.6%
S6 Heterotachy	Het	Terminal Clade Purifying	0.0%	0.0%	0.0%	0.0%	0.0%	0.0%
S6 Heterotachy	Neutral	Neutral Control	0.1%	0.0%	0.0%	0.0%	0.0%	0.0%
S8 Aromatic	Aromatic	F, Y, W Target (Unmodeled)	22.4%	0.2%	0.6%	0.3%	0.3%	0.4%
S8 Aromatic	NonArom	Non-aromatic Target	5.7%	0.4%	1.4%	0.9%	0.6%	0.7%
S8 Aromatic	Neutral	Neutral Control	1.7%	0.2%	0.2%	0.3%	0.1%	0.1%

Supplementary Notes

Computational Performance

We characterized the computational efficiency of the G-PRIME and E-PRIME implementations by analyzing the execution times for 18,944 mammalian genes. All analyses were performed on a standardized high-performance computing environment utilizing 4 cores of Ampere Altra (Neoverse-N1) ARM 128-core processors running at 3GHz. These benchmarks demonstrate that while the biophysical realism of PRIME requires more computational effort than property-agnostic models, the implementations remain practical for large-scale genomic applications. The G-PRIME model, which estimates gene-wide property weights, exhibits a mean execution time of 42.1 ± 82.2 minutes per gene. Over 75% of the mammalian alignments were processed in under 35 minutes, confirming that G-PRIME is suitable for routine gene-level characterization. These runtimes include LRT testing for each of the five individual property weights. The episodic E-PRIME model, which utilizes random-effects mixture modeling to capture fluctuating biophysical pressures, exhibits significantly higher computational demands. E-PRIME yielded a mean runtime of 4.57 ± 7.7 hours per gene, with a median of 2.36 hours. These runtimes also include LRT testing for each of the ten individual property weights (once per component). These results underscore that while S-PRIME and G-PRIME provide efficient site-level and gene-level resolution, the full characterization of episodic biophysical selection across the proteome necessitates substantial high-performance computing resources, totaling approximately 350,000 CPU-hours for the 120-mammal dataset.

Implementation and Availability

The S-PRIME framework is implemented as a standard analysis within the HyPhy software package (version 2.5.94 or later). The G-PRIME and E-PRIME models are implemented using the `FitModel` analysis available in the `hyphy-analyses` repository (`https://github.com/veg/hyphy-analyses`).

Supporting scripts used for the analyses presented in this manuscript, including the mammalian genome screen, simulation pipelines, structural mapping, and DMS validation, are available in a dedicated reproducibility repository at `https://github.com/veg/PRIME`.

Running PRIME Models in HyPhy

PRIME models can be invoked via the HyPhy command-line interface. By default, the models utilize the 5 physicochemical properties discussed in this manuscript: Hydrophobicity, Volume, Isoelectric Point, Alpha-Helix Propensity, and Beta-Sheet Propensity. Users may also provide custom property vectors or select from alternative sets (e.g., Atchley factors) during the analysis setup.

G-PRIME (Global Model) Gene-wide physicochemical constraints are estimated using the `FitModel.bf` analysis with the `MG_REV_PROPERTIES` model. This analysis is available in the `hyphy-analyses` repository:

```
hyphy [path_to_hyphy-analyses]/FitModel/FitModel.bf
      --alignment [path_to_msa]
      --model MG_REV_PROPERTIES
      --property-set 5PROP
```

E-PRIME (Episodic Model) Episodic property selection is detected using the `FitModel.bf` analysis with the `MG_REV_PROPERTIES_BSREL` model:

```
hyphy [path_to_hyphy-analyses]/FitModel/FitModel.bf
      --alignment [path_to_msa]
      --model MG_REV_PROPERTIES_BSREL
      --property-set 5PROP
```

S-PRIME (Site-level Model) Site-specific biophysical drivers are resolved using the dedicated `prime` analysis built into HyPhy:

```
hyphy prime --alignment [path_to_msa] --property-set 5PROP
```

Standard Output

All PRIME implementations generate a JSON-formatted result file containing maximum likelihood estimates for property weights (λ), model fit statistics (log-likelihood, AICc), and significance test results (LRT p-values). These files are designed for downstream visualization and integration into bioinformatics pipelines.

Supplementary Note: Statistical Determinants of S-PRIME Significance

To explicitly resolve the statistical determinants of physicochemical constraint, we performed a logistic regression analysis on 4,558 variable sites (substitutions > 0) to predict the probability of identifying significant structure (Omnibus $q \leq 0.10$). We modeled this probability as a function of the number of inferred substitutions, the amino acid diversity, and their interaction, while controlling for synonymous (α) and non-synonymous (β) rates. Our analysis reveals that PRIME significance is not merely a proxy for rapid evolution. While the raw number of substitution events is the strongest positive predictor (Odds Ratio ≈ 49 per SD increase, $p < 10^{-100}$), this effect is modulated by a critical negative interaction with amino acid diversity (Interaction Coef = -2.13, $p < 10^{-13}$). This result underscores a fundamental property of the framework: for a fixed number of evolutionary events, the probability of detecting significant structure *decreases* as the site explores a broader range of the amino acid state space. Furthermore, sites with higher non-synonymous rates (β) are significantly *less* likely to be identified as structured (Coef = -0.85, $p = 0.01$) once event counts are controlled for, mathematically distinguishing property-driven selection from generic rate acceleration.

These statistical fingerprints demonstrate that PRIME specifically targets toggling regimes—sites where frequent evolutionary turnover is strictly confined to a small physicochemically constrained subset of amino acid substitutions. For instance, Site 184 in HIV-RT exhibits 46 inferred substitutions yet remains restricted to just 3 amino acids (M, V, I). PRIME correctly identifies this as a highly structured regime ($Q < 10^{-6}$), driven by the strict conservation of hydrophobicity ($\lambda = 15.0$). In stark contrast, Site 75 in Camelid VHH undergoes 21 substitutions and explores 9 different amino acids. Although standard methods identify this site as undergoing significant diversifying selection (MEME $p = 0.037$), PRIME assigns it a non-significant weight ($Q = 0.53$). Structural analysis confirms this residue is highly solvent-exposed (RSA = 0.58), characterizing it as unconstrained diversification rather than the specific structural toggling observed at Site 184. However, with sufficient evolutionary information (e.g., > 50 substitutions), PRIME can recover subtle physicochemical biases even in highly variable sites (e.g., Camelid VHH Site 53, 18 amino acids, $Q = 0.006$), suggesting that weak but persistent structural preferences shape even the most hypervariable regions. Even in regimes of low turnover, PRIME can detect significant constraints if the substitutions are highly specific. For example, HIV-RT Site 151 (a known drug resistance position) undergoes only 4 inferred substitutions but is identified as highly significant ($Q < 10^{-6}$), driven by a specific toggle between two amino acid states that modulates hydrophobicity ($\lambda = -4.6$) while strictly conserving charge.

Empirically, our benchmark suggests that PRIME effectively resolves constraints when sites exhibit this toggling behavior: for positions restricted to 2 or 3 amino acid states, detection power exceeds 65% with as few as 6–10 substitutions. Conversely, highly variable sites exploring broad chemical space (> 8 amino acid states) generally require substantial evolutionary turnover (> 20 substitutions) to distinguish specific structural preferences from neutral drift.

To explore how rate-based selection (dN/dS) relates to biophysical constraints, we compared site-specific FEL rates (ω) to minimum and maximum S-PRIME property weights (λ) across three site classes (Supplementary Figure S2). Across all variable sites ($\omega \geq 0.1$, $N = 3,655$), ω is uncorrelated with the most diversifying weight ($\min(\lambda)$, Spearman $\rho = 0.052$) or the most conserving weight ($\max(\lambda)$, Spearman $\rho = -0.063$), indicating global decoupling at the site level (Supplementary Figure S2A). However, at mutually significant positive selection sites (FEL $p \leq 0.05, \omega > 1$ and S-PRIME $q \leq 0.10$, $N = 48$), higher ω correlates with both stronger diversifying selection ($\min(\lambda)$, $\rho = -0.300, p = 0.038$) and stronger purifying conservation ($\max(\lambda)$, $\rho = 0.339, p = 0.018$) on different properties (Supplementary Figure S2B). This indicates channeled positive selection, where rapid adaptation along one biophysical axis occurs against a background of purifying conservation on others. Conversely, at mutually significant purifying sites (FEL $p \leq 0.05, \omega < 1$ and S-PRIME $q \leq 0.10$, $N = 224$), property-specific constraints weaken as overall purifying selection relaxes (higher ω), showing a positive correlation for $\min(\lambda)$ ($\rho = 0.243, p = 2.5 \times 10^{-4}$) and a negative correlation for $\max(\lambda)$ ($\rho = -0.150, p = 0.025$; Supplementary Figure S2C).

References

Anisimova, M. and Yang, Z. 2004. Molecular evolution of the hepatitis delta virus antigen gene: recombination or positive selection? J Mol Evol, 59(6): 815–26.

Atchley, W. R., Zhao, J., Fernandes, A. D., and Drüke, T. 2005. Solving the protein sequence metric problem. Proceedings of the National Academy of Sciences of the United States of America, 102(18): 6395–6400.

Bush, R. M., Bender, C. A., Subbarao, K., Cox, N. J., and Fitch, W. M. 1999. Predicting the evolution of human influenza a. Science, 286(5446): 1921–5.

Dunn, K. A., Kenney, T., Gu, H., and Bielawski, J. P. 2019. Improved inference of site-specific positive selection under a generalized parametric codon model when there are multinucleotide mutations and multiple nonsynonymous rates. BMC Evol Biol, 19(1): 22.

Jarvis, E. D., Mirarab, S., Aberer, A. J., Li, B., Houde, P., Li, C., Ho, S. Y. W., Faircloth, B. C., Nabholz, B., Howard, J. T., Suh, A., Weber, C. C., da Fonseca, R. R., Li, J., Zhang, F., Li, H., Zhou, L., Narula, N., Liu, L., Ganapathy, G., Boussau, B., Bayzid, M. S., Zavidovych, V., Subramanian, S., Gabaldón, T., Capella-Gutiérrez, S., Huerta-Cepas, J., Rekepalli, B., Munch, K., Schierup, M., Lindow, B., Warren, W. C., Ray, D., Green, R. E., Bruford, M. W., Zhan, X., Dixon, A., Li, S., Li, N., Huang, Y., Derryberry, E. P., Bertelsen, M. F., Sheldon, F. H., Brumfield, R. T., Mello, C. V., Lovell, P. V., Wirthlin, M., Schneider, M. P. C., Prosdocimi, F., Samaniego, J. A., Vargas Velazquez, A. M., Alfaro-Núñez, A., Campos, P. F., Petersen, B., Sicheritz-Ponten, T., Pas, A., Bailey, T., Scofield, P., Bunce, M., Lambert, D. M., Zhou, Q., Perelman, P., Driskell, A. C., Shapiro, B., Xiong, Z., Zeng, Y., Liu, S., Li, Z., Liu, B., Wu, K., Xiao, J., Yinqi, X., Zheng, Q., Zhang, Y., Yang, H., Wang, J., Smeds, L., Rheindt, F. E., Braun, M., Fjeldsa, J., Orlando, L., Barker, F. K., Jønsson, K. A., Johnson, W., Koepfli, K.-P., O'Brien, S., Haussler, D., Ryder, O. A., Rahbek, C., Willerslev, E., Graves, G. R., Glenn, T. C., McCormack, J., Burt, D., Ellegren, H., Alström, P., Edwards, S. V., Stamatakis, A., Mindell, D. P., Cracraft, J., Braun, E. L., Warnow, T., Jun, W., Gilbert, M. T. P., and Zhang, G. 2014. Whole-genome analyses resolve early branches in the tree of life of modern birds. Science, 346(6215): 1320–31.

Kuno, G., Chang, G. J., Tsuchiya, K. R., Karabatsos, N., and Cropp, C. B. 1998. Phylogeny of the genus flavivirus. J Virol, 72(1): 73–83.

Martin, D. P., Weaver, S., Tegally, H., San, J. E., Shank, S. D., Wilkinson, E., Lucaci, A. G., Giandhari, J., Naidoo, S., Pillay, Y., Singh, L., Lessells, R. J., NGS-SA, COVID-19 Genomics UK (COG-UK), Gupta, R. K., Wertheim, J. O., Nekturenko, A., Murrell, B., Harkins, G. W., Lemey, P., MacLean, O. A., Robertson, D. L., de Oliveira, T., and Kosakovsky Pond, S. L. 2021. The emergence and ongoing convergent evolution of the sars-cov-2 n501y lineages. Cell, 184(20): 5189–5200.e7.

Mu, Y., Tian, R., Xiao, L., Sun, D., Zhang, Z., Xu, S., and Yang, G. 2021. Molecular evolution of tooth-related genes provides new insights into dietary adaptations of mammals. Journal of Molecular Evolution, 89(7): 458–471.

Nguyen, V. K., Su, C., Muyldermans, S., and van der Loo, W. 2002. Heavy-chain antibodies in camelidae; a case of evolutionary innovation. Immunogenetics, 54(1): 39–47.

Randall, J. G., Gatesy, J., and Springer, M. S. 2022. Molecular evolutionary analyses of tooth genes support sequential loss of enamel and teeth in baleen whales (mysticeti). Molecular Phylogenetics and Evolution, 171: 107463.

Rodrigue, N., Latrille, T., and Lartillot, N. 2021. A bayesian mutation-selection framework for detecting site-specific adaptive evolution in protein-coding genes. Mol Biol Evol, 38(3): 1199–1208.

Seoighe, C., Ketwaroo, F., Pillay, V., Scheffler, K., Wood, N., Duffet, R., Zvelebil, M., Martinson, N., McIntyre, J., Morris, L., and Hide, W. 2007. A model of directional selection applied to the evolution of drug resistance in hiv-1. Molecular Biology and Evolution, 24(4): 1025–1031.

Tamuri, A. U. and Dos Reis, M. 2022. A mutation-selection model of protein evolution under persistent positive selection. Mol Biol Evol, 39(1): msab309.

Wertheim, J. O., Murrell, B., Smith, M. D., Kosakovsky Pond, S. L., and Scheffler, K. 2015. Relax: detecting relaxed selection in a phylogenetic framework. Mol Biol Evol, 32(3): 820–32.

Yang, Z. 1998. Likelihood ratio tests for detecting positive selection and application to primate lysozyme evolution. Mol Biol Evol, 15(5): 568–73.

Yang, Z., Swanson, W. J., and Vacquier, V. D. 2000a. Maximum-likelihood analysis of molecular adaptation in abalone sperm lysin reveals variable selective pressures among lineages and sites. Mol Biol Evol, 17(10): 1446–55.

Yang, Z. H., Nielsen, R., Goldman, N., and Pedersen, A. M. K. 2000b. Codon-substitution models for heterogeneous selection pressure at amino acid sites. Genetics, 155: 431–449.

Yokoyama, S., Tada, T., Zhang, H., and Britt, L. 2008. Elucidation of phenotypic adaptations: Molecular analyses of dim-light vision proteins in vertebrates. Proc Natl Acad Sci U S A, 105(36): 13480–5.
